# Computational Studies on the Inhibitor Selectivity of Human JAMM Deubiquitinylases Rpn11 and CSN5

**DOI:** 10.3389/fchem.2018.00480

**Published:** 2018-10-09

**Authors:** Vikash Kumar, Michael Naumann, Matthias Stein

**Affiliations:** ^1^Institute of Experimental and Internal Medicine, Medical Faculty, Otto von Guericke University, Magdeburg, Germany; ^2^Molecular Simulations and Design Group, Max Planck Institute for Dynamics of Complex Technical Systems, Magdeburg, Germany

**Keywords:** computational drug design, deubiquitinase (DUB), selectivities, protein-protein interaction, molecular dynamics (MD), ligand binding

## Abstract

Deubiquitinylases (DUBs) are highly specialized enzymes which are responsible for removal of covalently attached ubiquitin(s) from the targeted proteins. DUBs play an important role in maintaining the protein homeodynamics. Recently, DUBs have emerged as novel therapeutic targets in cancer, inflammation, diabetes, and neurodegeneration. Among the different families of DUBs, the metalloprotease group or JAB1/MOV34/MPR1 (JAMMs) proteases are unique in terms of catalytic mechanism. JAMMs exhibit a Zn^2+^-dependent deubiquitinylase activity. Within the JAMM family, deubiquitinylases Rpn11 and CSN5 are constituents of large bimolecular complexes, namely the 26S proteasome and COP9 signalosome (CSN), respectively. Rpn11 and CSN5 are potential drug targets in cancer and selective inhibitors of both proteins have been reported in the literature. However, the selectivity of JAMM inhibitors (capzimin for RPN11 and CSN5i-3 for CSN5) has not been structurally resolved yet. In the present work, we have explored the binding modes of capzimin and CSN5i-3 and rationalize their selectivity for Rpn11 and CSN5 targets. We found that capzimin interacts with the active site Zn^+2^ of Rpn11 in a bidentate manner and also interacts with the residues in the distal ubiquitin binding site. MD simulations studies and binding energy analysis revealed that the selective binding of the inhibitors can be only explained by the consideration of larger heterodimeric complexes of Rpn11 (Rpn8-Rpn11) and CSN5 (CSN5-CSN6). Simulation of these protein-protein complexes is necessary to avoid unrealistic large conformational changes. The selective binding of inhibitors is mainly governed by residues in the distal ubiquitin binding site. This study demonstrates that selective inhibitor binding design for Rpn11 and CSN5 JAMM proteases requires consideration of heterodimeric protein-protein target structures.

## Introduction

Ubiquitinylation is one of the major post-translational modifications of proteins. Ubiquitin is a 76 amino acids protein which is covalently attached to the lysine residue of the substrate by consecutive action of three enzymes i.e., activating (E1), conjugating (E2), and ligating (E3) enzymes (Nandi et al., 2006). Ubiqutinylated proteins participate in various cellular processes. In order to remove the ubiquitinylation mark, separate families of proteins exist and they have been named as deubiquitinylases. In contrast to ubiquitinylation, deubiquitinylation requires action of a single enzyme only (Reyes-Turcu et al., 2009). DUBs have been classified into 5 families i.e., ubiquitin specific proteases (USPs), ovarian tumor proteases (OTUs), Machado-Joseph disease proteases (MJDs), and JAB1/MOV34/MPR1 (JAMM) proteases (Komander et al., 2009; Mevissen and Komander, 2017). Except for JAMM proteases which have Zn^2+^ in the catalytic site, all other DUBs are cysteine proteases. Members of JAMM family have a common Zn^2+^ binding motif which contains three conserved residues (one aspartate and two histidines) (Berndt et al., 2002).

The human genome encodes for 14 JAMM proteins and only seven of them have the full set of conserved residues required for Zn^+2^ binding (Shrestha et al., 2014). Six JAMM proteins have deubiquitinylase activities which are AMSH, AMSH-LP, BRCC36, CSN5, MYSM1, and Rpn11 (also known as POH1 or PSMD14) (Shrestha et al., 2014). CSN5 is a component of the CSN and it also possesses nedylation activity (Lee et al., 2011; Echalier et al., 2013). Monomeric CSN5 shows no DUB activity and requires the presence of its non-active binding partner (CSN6) to gain full activity (Echalier et al., 2013). Rpn11 is a component of the 19S regulatory subunit of the large proteasome complex and only displays deubiquitinylase activity (Verma et al., 2002). Similar to the CSN5, a monomeric Rpn11 is not catalytically active (Yao and Cohen, 2002; Pathare et al., 2014).

**Figure 1:**
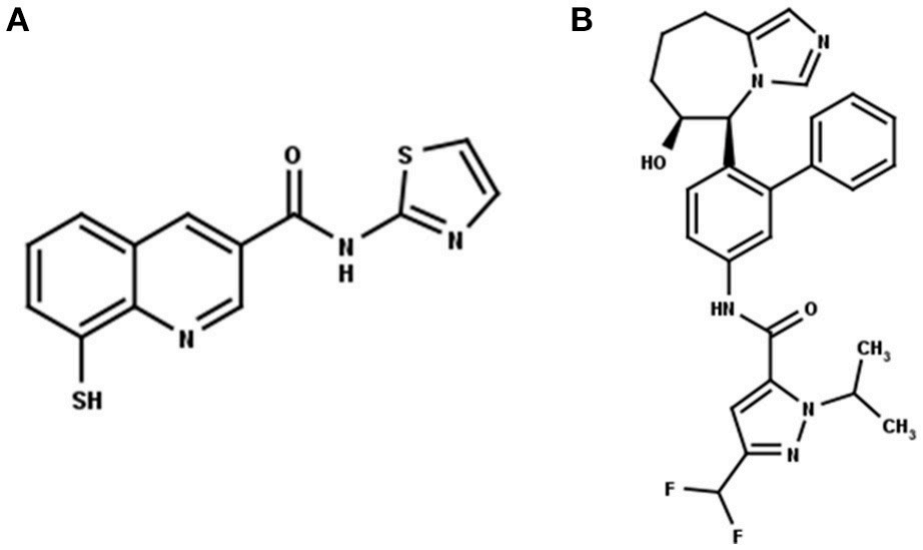
2D structure of the Rpn11 selective inhibitor, capzimin **(A)** and the CSN5 selective inhibitor, CSN5i-3 **(B)**. Both are targeting JAMMs but their selectivity of action cannot be rationalized.

Recently, Rpn11 has emerged as a potential drug target in human cancers (Li et al., 2017). Rpn11 is responsible for deubiquitinylation of proteasomal substrates (Verma et al., 2002) Inhibition of Rpn11 has been reported to overcome bortezomib resistance and induce apoptosis in multiple myeloma cells (Song et al., 2017). Capzimin is a potent and selective inhibitor of Rpn11 (Figure 1A) and it has been suggested that capzimin chelates the Zn^2+^ ion in the active site of Rpn11 (Li et al., 2017; Perez et al., 2017). Capzimin shows an 80-fold selectivity for Rpn11 over CSN5 (Li et al., 2017). Similarly, CSN5i-3, a potent inhibitor of CSN5 (Figure 1B), shows 10,000-fold selectivity for CSN5 over Rpn11. However, the structural basis of these inhibitor selectivities is not known. From the drug design perspective, it is important to rationalize the binding mode of capzimin and structural elements responsible for imparting DUB selectivity. In the present study, we used a workflow of molecular docking, refinement and ligand binding stability studies by molecular dynamics simulations and binding energy calculations to investigate the structural basis of selective inhibition of Rpn11 and CSN5.

## Materials and methods

### Monomer and heterodimeric target structures

**Figure 2:**
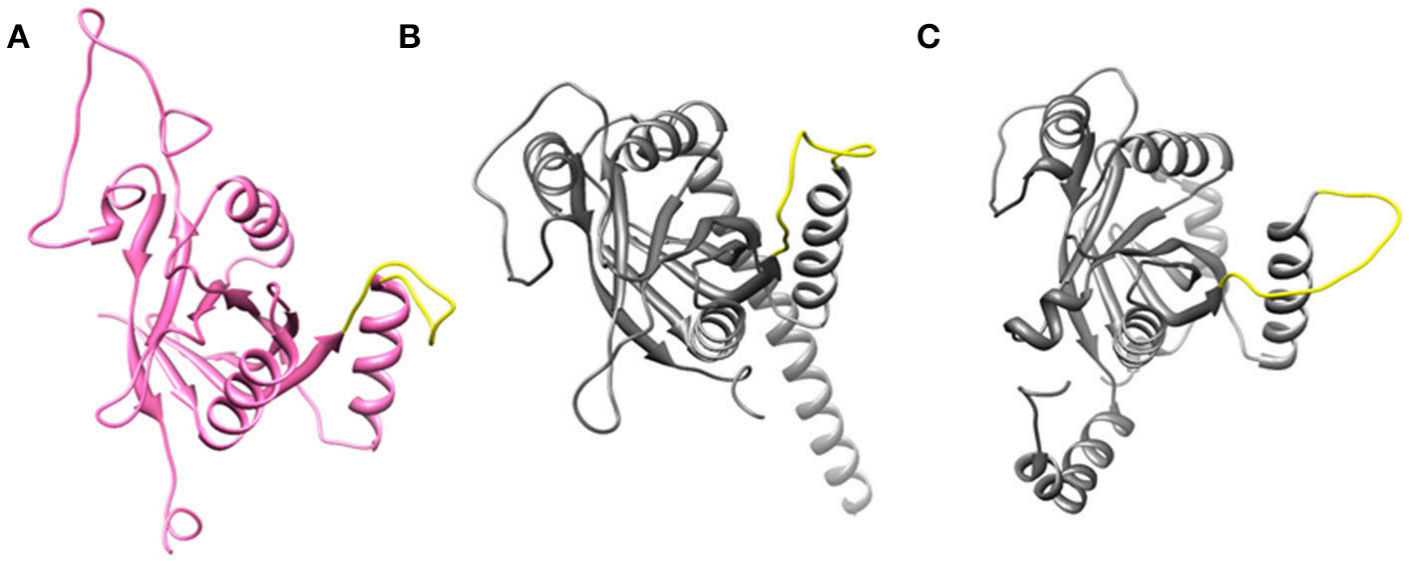
Open conformation of Ins-1 loop (yellow) in **(A)** Rpn11 monomer **(B)** CSN5 (monomeric state) and **(C)** CSN5 (as in heterodimeric state).

**Figure 3:**
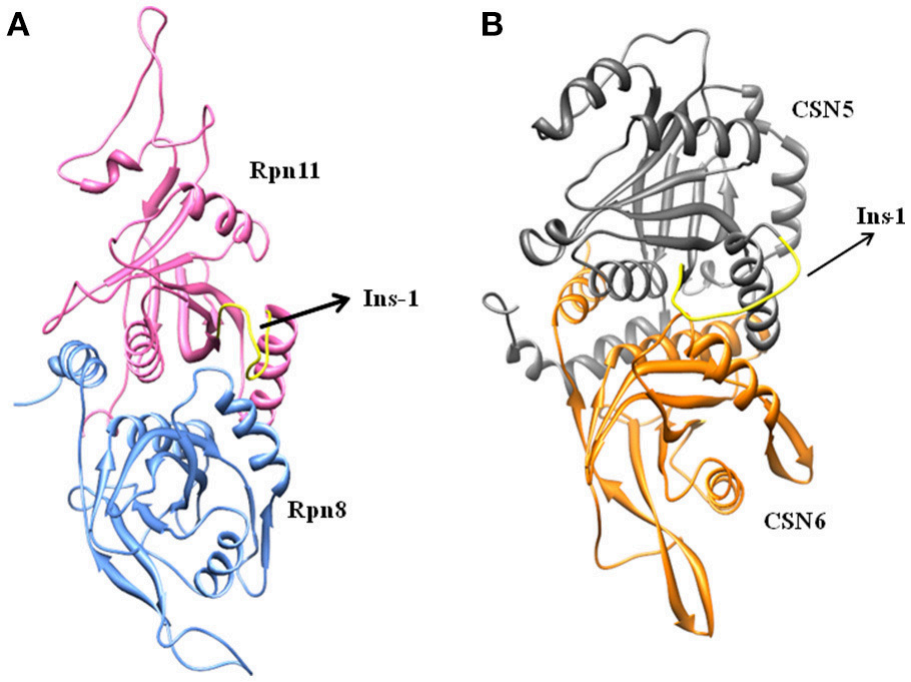
Processed structures of **(A)** Rpn8-Rpn11 heterodimer and **(B)** CSN5-CSN6 heterodimer.

Cryo-EM structure of human 26S proteasome (PDB_code: 5GJR) was obtained from PDB (Huang et al., 2016). With the exception of the chains U (Rpn8 or PSMD7) and V (Rpn11 or PSMD14), other were deleted. The Rpn-Rpn11 heterodimer in the unprocessed form is shown in Figure S1A. We noticed that RPN11 does not have Zn^2+^ ion in the catalytic site. Rpn8 and Rpn11 were processed separately. We deleted the C-terminal region of Rpn8 (182–295) and Rpn11 (210–316) since they are not relevant for enzymatic catalysis. With the help of UCSF Chimera (Pettersen et al., 2004) interface to MODELLER (Sali and Blundell, 1993; Webb and Sali, 2017), missing regions in Rpn8 (143–151) and Rpn11 (164–189) were modeled as loops (Figure 2A and Figure S1). Ins-1 loop (76–88) of the Rpn11 was initially in the closed conformation hence we generated 10 alternative conformations of Ins-1 loop (Figure S1B). We selected a loop conformation which was pointing away from the catalytic site (Figure 2A). The final Rpn8-Rpn11 heterodimer model is shown is Figure 3A and Figure S1C. A Zn^2+^ ion was transferred to the catalytic site of Rpn11 by aligning the Rpn11 structure with the crystal structure of CSN5. After transferring Zn^2+^ ion to the Rpn11, we used both the Rpn11 monomer (Figure 2A) and the Rpn8-Rpn11 heterodimer (Figure 3A) structures for subsequent analysis.

For the CSN5 monomer (Figure 2B), the recently solved crystal structure of CSN5 with CSN5i-3 (PDB_code: 5JOG) (Schlierf et al., 2016) was considered. We modeled the missing Ins-1 loop region (Figure S2A) by analogy to Rpn11 (see above) and out of 5 loop conformations (Figure S2B), a loop conformation which did not show any steric clash and contact with the bound inhibitor was considered (Figure S2C). The structure of the CSN5-CSN6 heterodimer (Figure S3A) was extracted from the crystal structure of human COP9 signalasome (PDB_code: 4D10) (Lingaraju et al., 2014) C-terminal regions of CSN5 (258–333) and CSN6 (215–316) were also deleted since they are not involved in catalysis and heterodimer formation can take place without them. Here, Ins-1 loop (98–113) of CSN5 was found to be in a closed conformation. We generated 10 alternative conformations of Ins-1 loop (Figure 2C and Figure S3B). We eventually selected a CSN5 model in which the Ins-1 loop is in an open conformation and does not obstruct ligand access to the catalytic site. The processed CSN5-CSN6 heterodimer is shown in Figure 3B and Figure S3C.

### Inhibitor positioning

The 3D structure of capzimin was generated with the help of MarvinSketch program (https://chemaxon.com/products/marvin). The sulfur thiolate has a negative charge. Ligand docking of capzimin was carried out with AUTODOCK4.2 (Morris et al., 2009) Kollman charges were assigned to the atoms of Rpn11 and CSN5. On Zn, +2 charge was manually assigned. Partial charges on capzimin were calculated using the SwissParam server (Zoete et al., 2011). For both Rpn11 and CSN5, a grid was centered on the catalytic Zn^2+^. To enclose the binding site in RPN11, the size of the 3D grid was set to 46, 50, and 52 grid points in x, y and z directions, respectively, with a default spacing of 0.375 Å. In case of CSN5, binding site was enclosed in a grid consisting of 48, 50, and 64 grid points in x, y and z directions, respectively. In each case, the Lamarckian Genetic Algorithm (LGA) was used to generate 100 docked conformations of capzimin. Binding mode of CSN5i-3 in the CSN5 monomer is known (Schlierf et al., 2016) hence we used the co-crystalized conformation of CSN5i-3 to generate CSN5i-3 bound CSN5-CSN6 heterodimeric, Rpn11 monomeric and Rpn8-Rpn11 heterodimeric complexes by manual docking (structural superimposition).

### Molecular dynamics simulations

All MD simulations were carried out using GROMACS-5.1.2 (Van Der Spoel et al., 2005). For monomeric Rpn11, CSN5, and heterodimeric complexes Rpn11-Rpn8 and CSN5-CSN6 the all-atom CHARMM27 force field (which has CHARMM22 and CMAP for proteins) (Mackerell et al., 1998, 2004) provided in the GROMACS package was used. CHARMM27 force field provides non-bonded parameters for Zn^2+^. Optimized forcefield parameters for Zn^+2^ were taken from Stote and Karplus (1995) and were shown to give reliable coordination geometries. The topology files for capzimin and CSN5i-3 (see Supplementary File for more information) were generated with the help of the SwissParam server (Zoete et al., 2011). All complexes were enclosed in triclinic boxes (see Table S1). The TIP3P water (Jorgensen, 1981; Mark and Nilsson, 2001) model was used to solvate all complexes. Ions (Na^+^ and Cl^−^) were added at 0.15 M concentration to neutralize the systems. After neutralization, the systems were subjected to 5000 steps of steepest decent minimization. Minimized systems were further equilibrated under both NVT and NPT conditions for 1 and 2 ns, respectively. During equilibration, position restrains were applied to both protein (including Zn^+2^) and ligand atoms. Temperature (310 K) and pressure (1 atm) were controlled by the velocity rescaling thermostat (Bussi et al., 2007) and Parrinello-Rahman barostat (Parrinello and Rahman, 1981), respectively. The equilibrated systems were finally subjected to the 100 ns production phase under NPT condition without any position restraints. Three independent simulations were carried out for each of the complexes.

### Binding energy calculation

Binding energies of inhibitors were calculated with the help of the linear interaction energy (LIE) methodology. LIE methodology has been reported to predict reliable binding energies (Hansson et al., 1998; Aqvist and Marelius, 2001). In the LIE methodology, the free energy of transfer of ligand from water to the protein environment is giving the binding energy. In simple terms, the LIE equation can be given as:

(1) $$\Delta {\text{G}}_{\text{bind}}\;(\text{ligand})={\text{G}}^{\text{bound}}{}_{\text{sol}}\;(\text{ligand})-{\text{G}}^{\text{free}}{}_{\text{sol}}\;(\text{ligand})$$

Where ΔG_bind_ (ligand) is binding energy of ligand, ${\text{G}}_{\text{sol}}^{\text{bounds}}$ (ligand) is the energy of ligand in the solvated protein-ligand complex and ${\text{G}}_{\text{sol}}^{\text{free}}$ (ligand) is the energy of the free ligand in water. LIE calculation is generally carried out in combination with MD or Monte Carlo (MC) simulation. LIE has two components, i.e., electrostatic (el) and van der Waals (vdW) interactions^30^. Hence, Equation (1) can be rewritten as

(2) $$\begin{array}{l} \Delta {\text{G}}_{\text{bind}}=\alpha ({\langle {\text{U}}_{\text{lig-surr}}^{\text{vdW}}\rangle }_{\text{protein }}-{\langle {\text{U}}_{\text{lig-surr}}^{\text{vdW}}\rangle }_{\text{water }})\\ \;+\beta ({\langle {\text{U}}_{\text{lig-surr}}^{\text{el}}\rangle }_{\text{protein }}-{\langle {\text{U}}_{\text{lig-surr}}^{\text{el}}\rangle }_{\text{water }})\\ \;=\alpha \Delta {\text{U}}^{\text{vdW}}+\beta \Delta {\text{U}}^{\text{el}} \end{array}$$

Where brackets < > indicate thermodynamic averages of the interaction energies of the ligand with its surroundings (Aqvist and Marelius, 2001). α is an empirically derived non-polar scaling factor and β is a polar scaling factor (Aqvist and Marelius, 2001). Almlöf et al. have suggested β_0_ = 0.43 for neutral compounds and correction factors (Δβ) for different ligands (Almlöf et al., 2007). For calculation of ligand binding energies we have used α = 0.18. β values for the thiolate form of capzimin (anion) and CSN5i-3 (alcohol) were obtained after applying functional group-specific correction factors to the β_0_ (0.43+0.02 = 0.45 for capzimin and 0.43–0.06 = 0.37 for CSN5i-3) (Almlöf et al., 2007; Gutiérrez-De-Terán and Aqvist, 2012). Values of β depend on the chemical nature of the ligand (Hansson et al., 1998; Rinaldi et al., 2004). In order to calculate the energy terms (vdW and el) of capzimin and CSN5i-3 in the water, we have carried out separate 50 ns MD simulation for each.

## Results and discussion

### Open conformations of Rpn11 and CSN5

Ins-1 region of CSN5 (97–131) and RPN11 (74–106) has been reported to be flexible and its flexibility is important for the binding of distal ubiquitin (Echalier et al., 2013; Worden et al., 2014). In the cryo-EM structure of Rpn11, the Ins-1 loop (76–88) obstructs the distal ubiquitin binding pocket. Previous studies suggest that the Ins-1 loop is flexible and very important for the regulation of enzymatic activity of zinc metalloproteases. It seems that during ligand binding this Ins-1 loop can adopt different conformations. Keeping the above fact in mind, we generated alternative loop conformations so that the distal ubiquitin binding pocket becomes accessible for the binding of inhibitors. In the open conformation, Ins-1 loop is pointing away from the catalytic site (Figure 2). We have used two different crystal structures to represent CSN5 in monomeric and heterodimeric states. In the monomeric state, CSN5 is already complexed with a potent inhibitor CSN5i-3 and a part of Ins-1 loop (100–106) is missing but we have modeled the Ins-1 loop in open conformation. In the crystal structure of CSN5-CSN6 heterodimer, initial Ins-1 loop (98–109) conformation blocks the distal ubiquitin binding site which we also observed in cryo-EM structure of Rpn11. Hence, Ins-1 loop was remodeled in the open conformation (Figures 2B,C). Rpn8-RPN11 and CSN5-CSN6 heterodimeric states are shown in Figure 3.

### Initial docked conformations of capzimin

**Figure 4:**
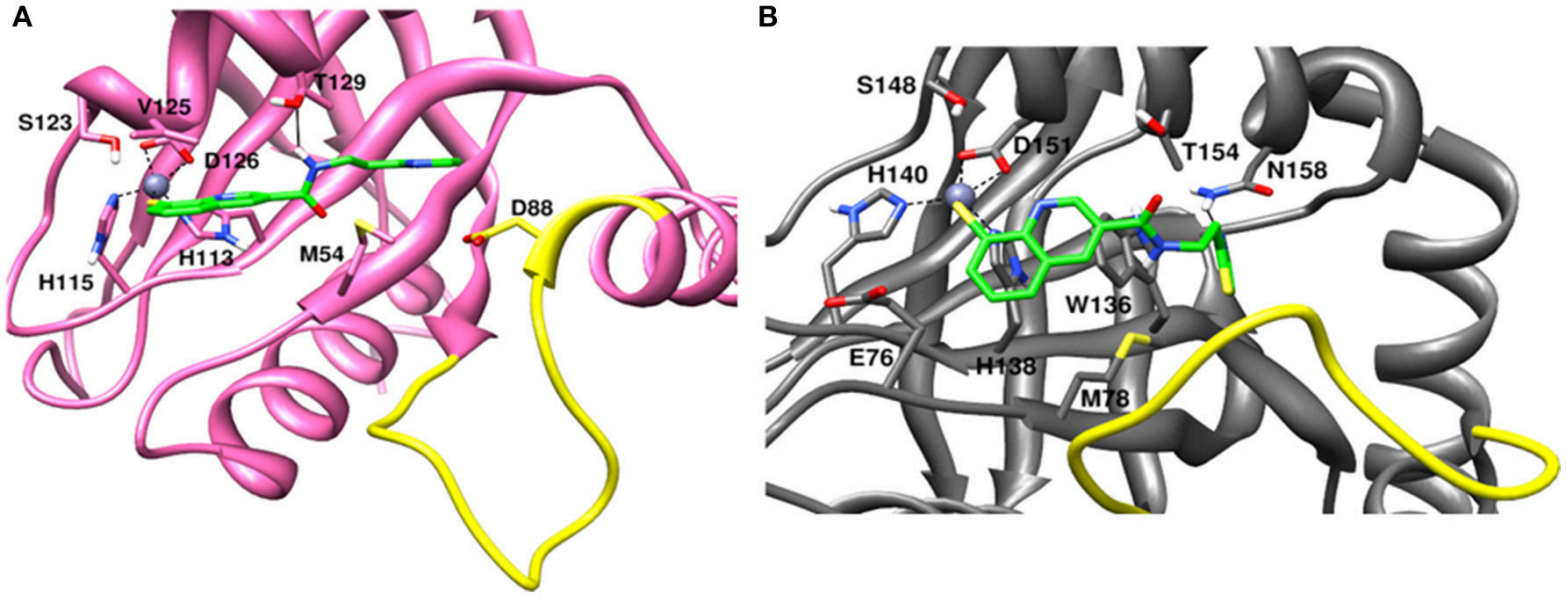
Docked conformation of capzimin in the **(A)** Rpn11 binding pocket and **(B)** CSN5 binding pocket.

The recently published crystal structure of CSN5 with CSN5i-3 provides a picture of inhibitor occupation of the distal ubiquitin binding site and possible interference with the catalytic activity of CSN5. Due to the conservation of residues in the distal ubiquitin binding site between CSN5 and Rpn11, we assumed that capzimin might also occupy the distal ubiquitin binding site of Rpn11. In the CSN5i-3 bound crystal structure of CSN5, the nitrogen atom of the azole ring makes a coordinate bond with Zn^2+^. In the previous study (Perez et al., 2017), the parent compound of capzimin that is 8-thiquinoline (8TQ) has been shown to interact with the Zn^2+^ in a bidentate manner and it has been proposed that capzimin inhibits Rpn11 also via chelation of the catalytic Zn^+2^. Interestingly, the top binding pose generated by AUTODOCK4.2 showed that the 8TQ moiety of capzimin also makes a monodentate interaction with Zn^2+^ of CSN5 and Rpn11 (Figure 4). Another possible coordinating atom of the 8TQ moiety was remote from the Zn^2+^ ion. The binding modes of capzimin in both Rpn11 and CSN5 appear similar but we observed few differences in the distal ubiquitin binding site. In RPN11, the amide portion of capzimin makes H bond with Thr129, and the thiazole moiety makes hydrophobic and van der Waals interactions with side chains of Met54 and Asp88, respectively (Figure 4A). In CSN5, H-bond with Thr154 was absent and thiazole moiety of capzimin makes H-bond with side chain of Asn158 and shows hydrophobic interaction with the sidechains of Met78 and Trp136 (Figure 4B).

### Binding of capzimin and CSN5i-3 to monomeric Rpn11 and Rpn8-Rpn11 heterodimer

**Figure 5:**
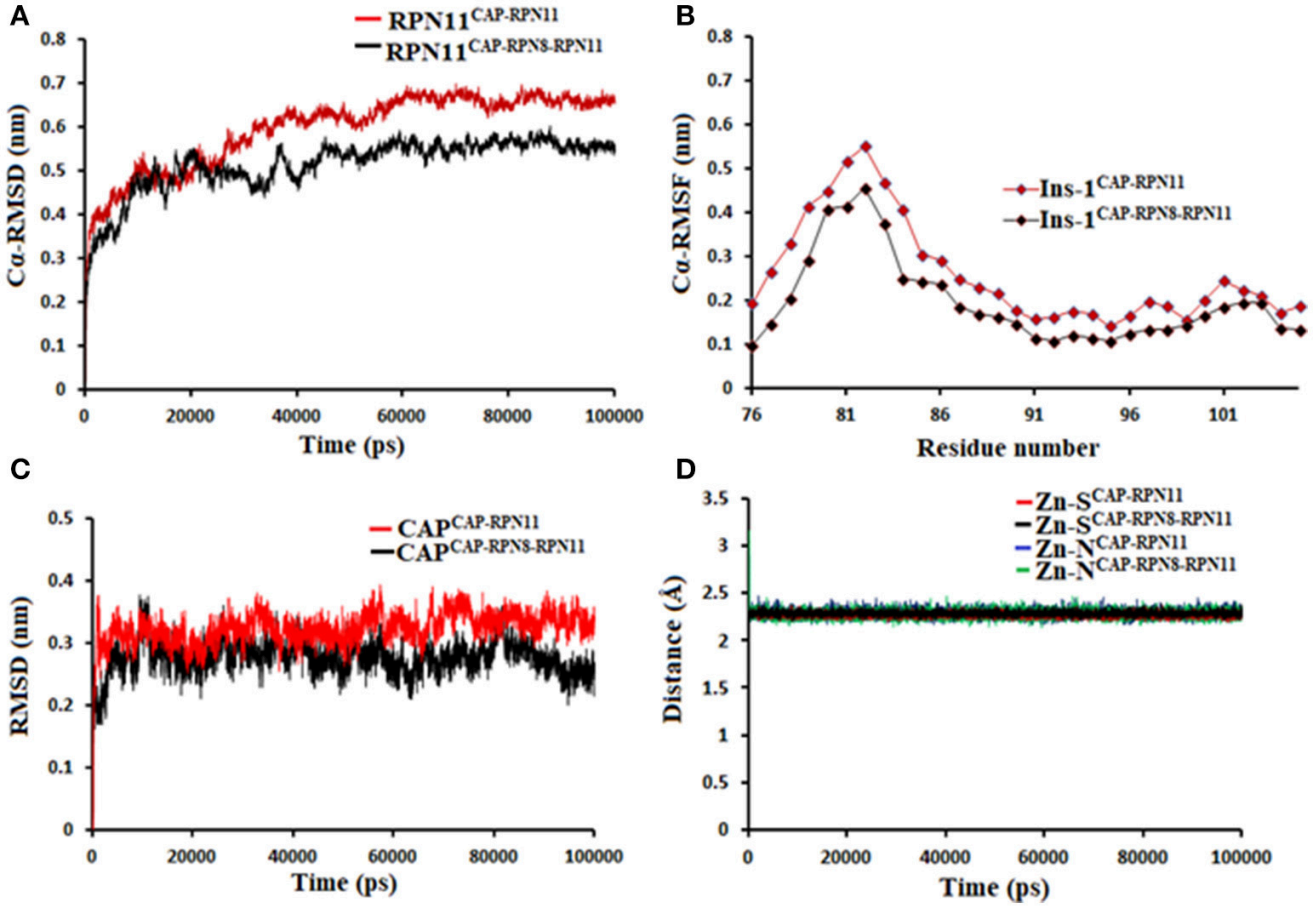
MD simulation data of Rpn11 bound to capzimin in the absence (red) and presence of Rpn8 (black). **(A)** Cα-RMSD of Rpn11 **(B)** Cα-RMSF of Ins-1 region of Rpn11 **(C)** RMSD of capzimin and **(D)** distances between coordinating atoms of capzimin and Zn^+2^. Zn^+2^-S distances in capzimin bound Rpn11 and Rpn8-Rpn11 heterodimer are shown in blue and green color, respectively.

**Figure 6:**
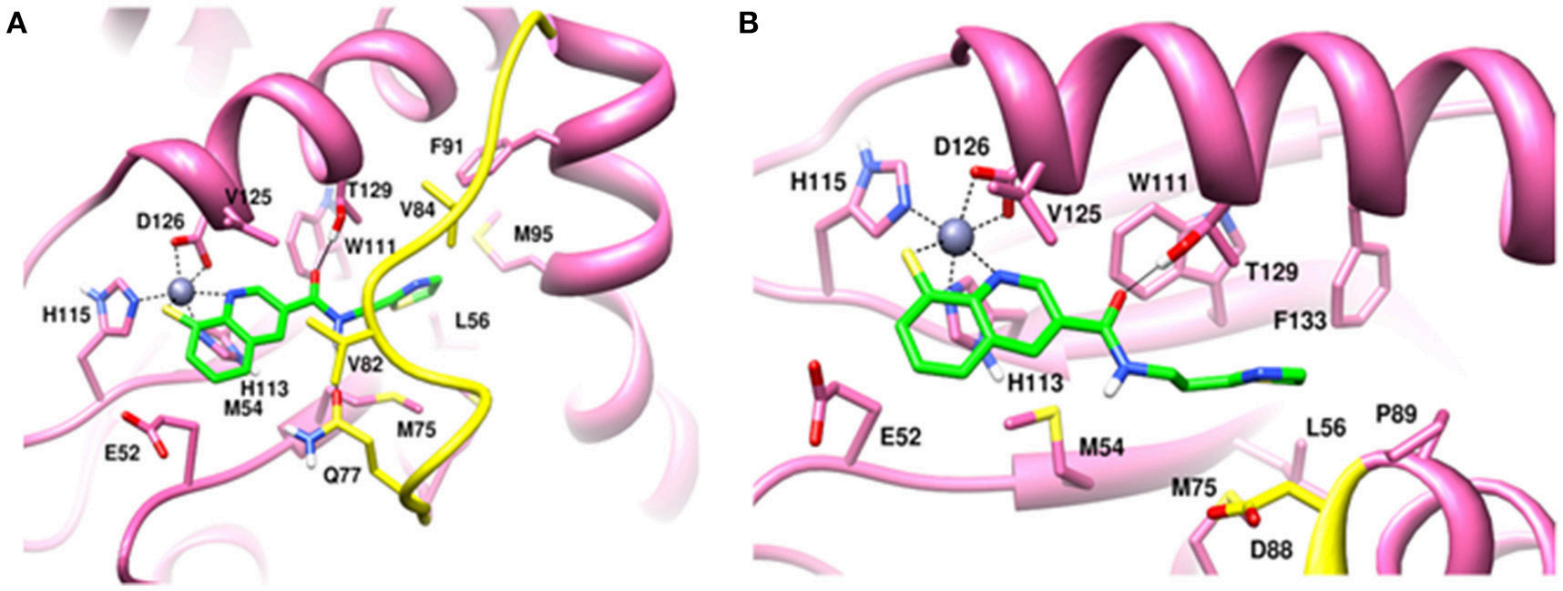
MD snapshots showing interaction of **(A)** capzimin with monomeric Rpn11 and **(B)** capzimin with Rpn11 in the presence of Rpn8. Yellow region corresponds to Ins-1 loop. H-bonds are represented by solid black line.

**Figure 7:**
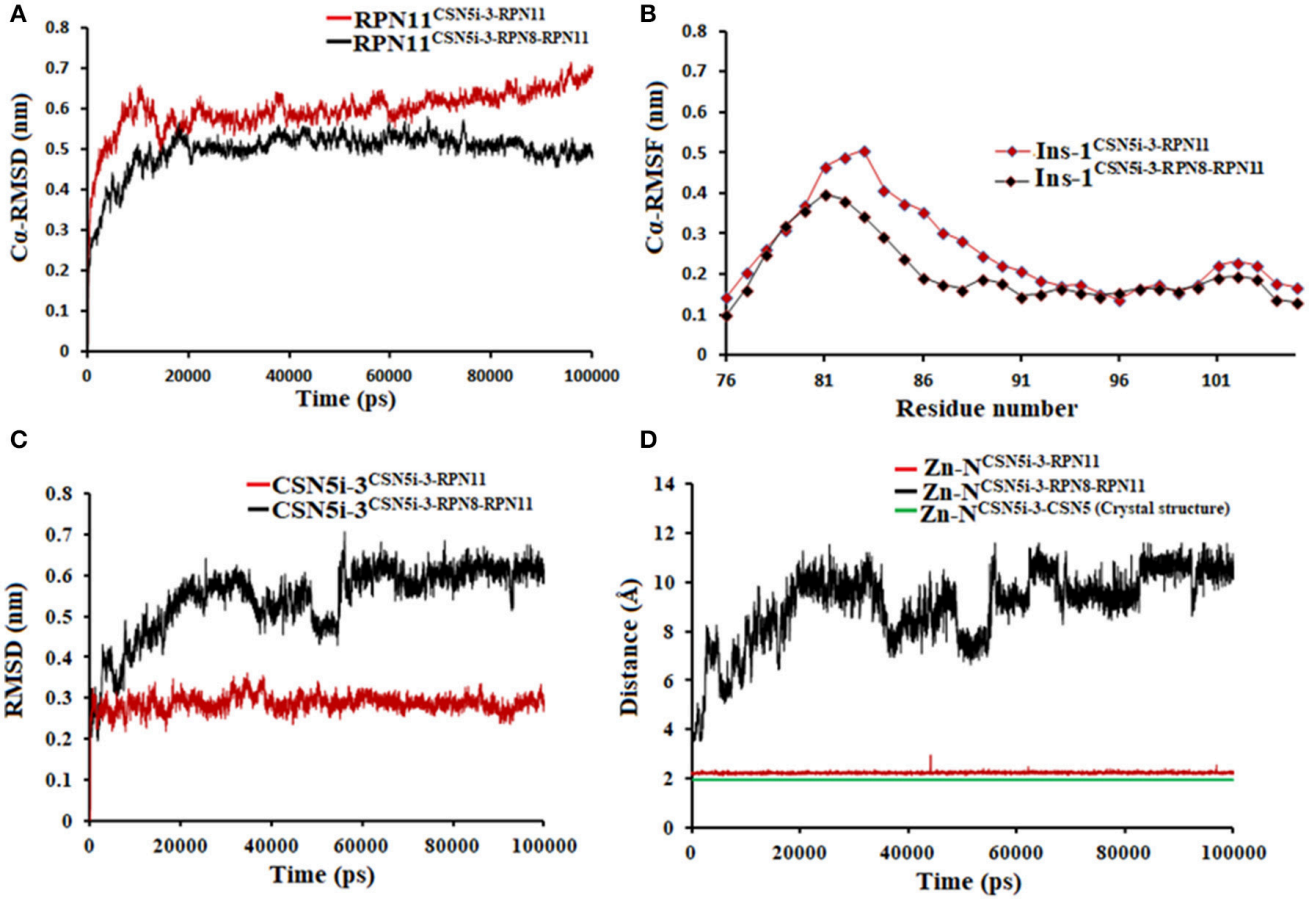
MD simulation data of Rpn11 bound to CSN5i-3 in the absence (red) and presence of Rpn8 (black). **(A)** Cα-RMSD of Rpn11 **(B)** Cα-RMSF of Ins-1 region of Rpn11 **(C)** RMSD of CSN5i-3 and **(D)** distances between coordinating atoms of CSN5i-3 and Zn^+2^. Zn^+2^-N distance in the crystal structure of CSN5i-3 bound CSN5 is shown in green color.

**Figure 8:**
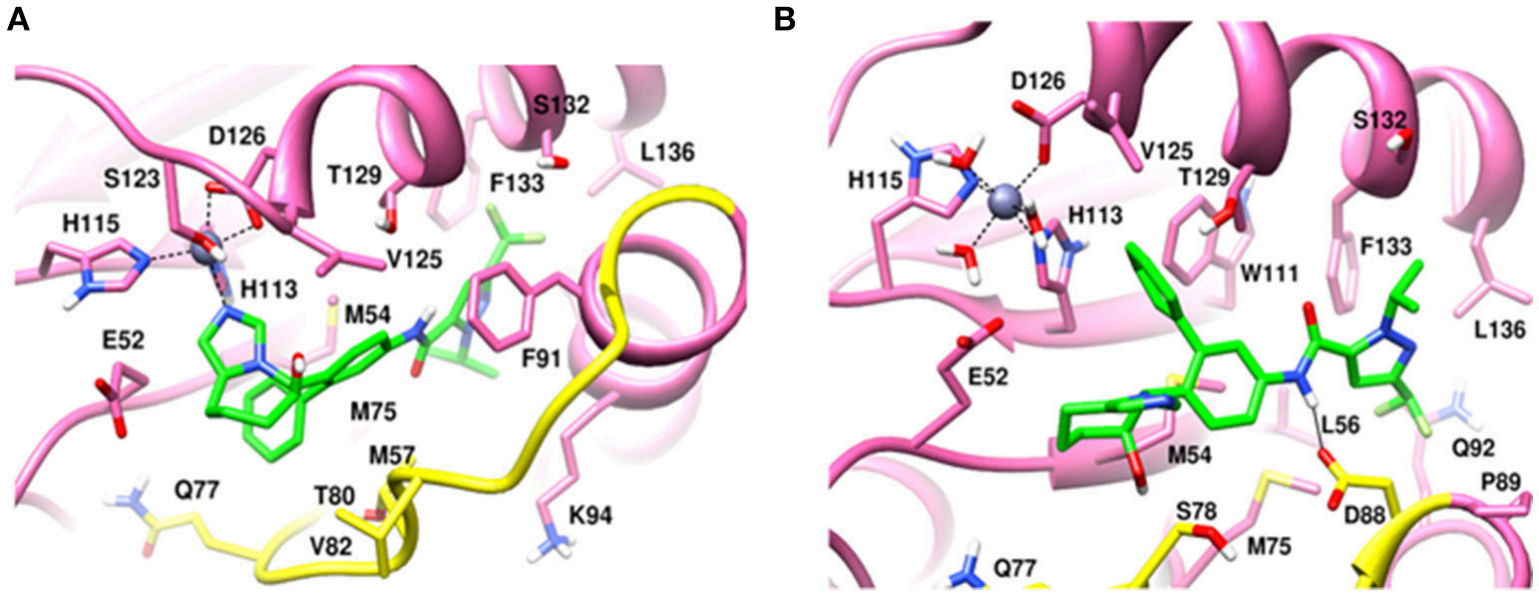
MD snapshots showing interaction of **(A)** CSN5i-3 with monomeric Rpn11 and **(B)** CSN5i-3 with Rpn11 in the presence of Rpn8. Yellow region corresponds to Ins-1 loop. H-bonds are represented by solid black line.

**Figure 9:**
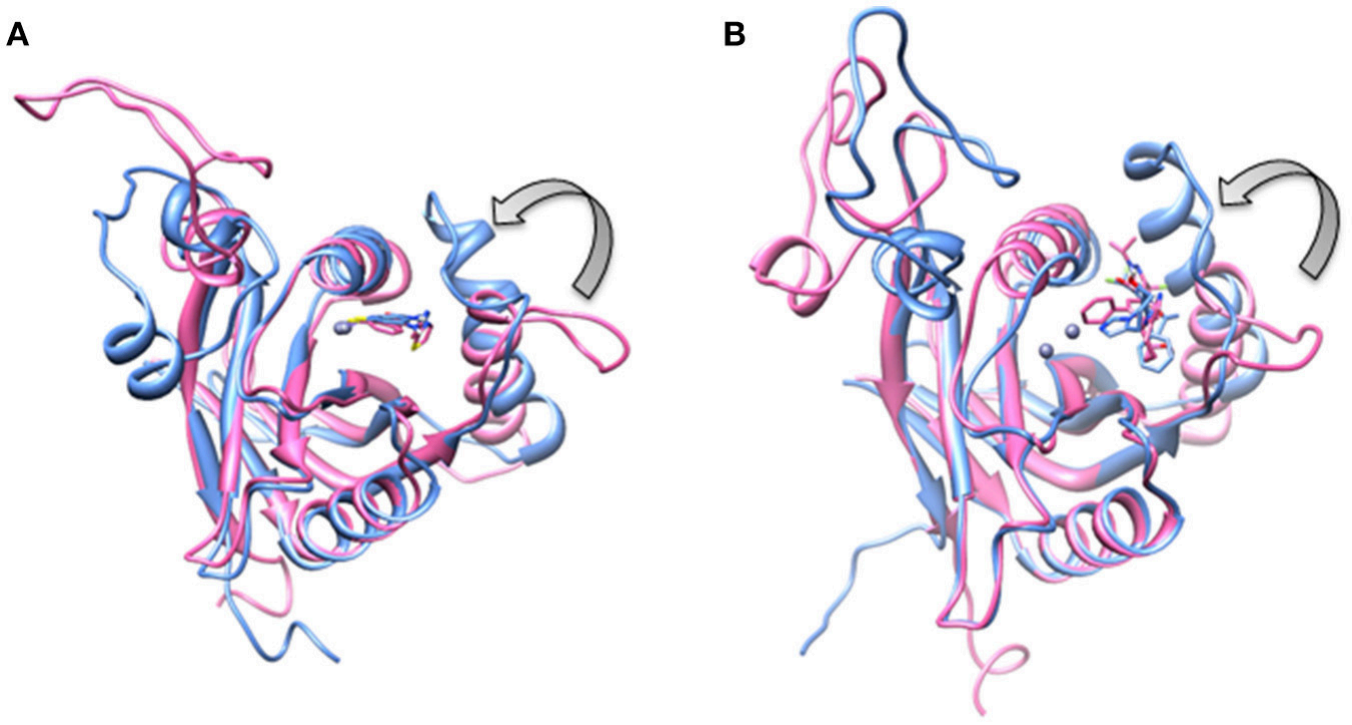
Aligned structures of inhibitors bound Rpn11 in the absence (blue) and presence of Rpn8 (hot pink) in which Ins-1 loop has moved (curved arrow) toward the distal ubiquitn binding site **(A)** capzimin bound Rpn11 and **(B)** CSN5i-3 bound Rpn11.

Being an integral part of the proteasome machinery, Rpn11 works in coordination with other subunits. Monomeric Rpn11 lacks deubiquitinylase activity and is active only in the presence of Rpn8. Here, we have investigated the binding of Rpn11 inhibitors in the absence and presence of Rpn8 (Figures 5–9). Cα root mean square deviation (Cα-RMSD) analysis revealed that compared to the monomer capzimin-Rpn11 complex, Rpn11 in the heterodimeric capzimin-Rpn8-Rpn11 complex shows lower RMSD (Figure 5A) and is thus stabilized. Cα root mean square fluctuation (Cα-RMSF plot) (Figure 5B) showed that in the absence of Rpn8, residues belonging to the Ins-1 loop and α2 helix undergo larger fluctuations. Structural analysis also revealed that in the capzimin-Rpn11 complex, the Ins-1 loop and α2 helix exhibited large movements. Particularly, the α2 helix came closer to the α3 helix (Figure 9A). The presence of Rpn8 restricts this movement of α2 helix as well as that of the Ins-1 loop. Both crystal structures of the yeast Rpn8-Rpn11 heterodimer (Pathare et al., 2014) and the cryo-EM structure of human 26S proteasome (Huang et al., 2016) show that the α2 helices of both proteins makes extensive contacts with each other.

Molecular dynamics simulations refined the binding position of capzimin. The average RMSDs of the refined structures to the starting were 0.32 and 0.27 Å in Rpn11 monomer and Rpn8-Rpn11 heterodimer, respectively. In the heterodimeric state, capzimin showed overall less deviation in (Figure 5C). It seems that during the start of simulation, capzimin tries to optimize interactions with the residues of the binding pocket and this leads to the deviation from initial conformation. However, in both complexes, S-Zn^2+^ and N-Zn^2+^ distances (Table 1) were stable (Figure 5D) and comparable to the experimentally determined distances reported in [(Tp^Me, Ph^)Zn(8TQ)] complex (Perez et al., 2017). The H-bond between capzimin and the side chain of Thr129 was maintained during most of the part of trajectory (Figures S4A,B). In Figures 6A,B, we see that the 8TQ fragment of the capzimin interacts with the catalytic Zn^+2^ and the additional amide moiety interacts with Thr129. Leu56, Pro89, and Phe133 provides hydrophobic interactions to the azole moiety of the capzimin. Capzimin showed almost similar binding affinity to monomeric Rpn11 as well as Rpn8-Rpn11 heterodimer (Table 2).

**Table 1:** Distances of coordinating atoms of capzimin and CSN5i-3 from catalytic Zn^+2^ of Rpn11 and CSN5.

	**Distance (Å)**	
**System**	**N-Zn^+2^**	**S-Zn^+2^**
Capzimin-Rpn11	2.28 ± 0.04	2.27 ± 0.02
Capzimin-Rpn8-Rpn11	2.27 ± 0.04	2.28 ± 0.02
Capzimin-CSN5	2.30 ± 0.07	2.30 ± 0.02
Capzimin-CSN5-CSN6	4.04 ± 0.14	2.28 ± 0.02
CSN5i-3-Rpn11	2.23 ± 0.04	
CSN5i-3-Rpn8-Rpn11	9.10 ± 1.51	
CSN5i-3-CSN5	2.22 ± 0.02	
CSN5i-3-CSN5-CSN6	2.18 ± 0.02	

*Data represented here are averages of three independent MD simulation runs*.

**Table 2:** Binding energies of ligands calculated using LIE method.

	**Binding energy (kcal/mole)**	
**System**	**Capzimin**	**CSN5i-3**
Rpn11	−37.815 ± 2.898	−5.401 ± 2.023
**Rpn8-Rpn11**	−**38.265** ± **3.006**	**0.103** ± **2.023**
CSN5	−36.724 ± 3.007	−6.043 ± 2.010
**CSN5-CSN6**	−**26.429** ± **3.054**	−**8.710** ± **1.975**

*The final 20 ns of each trajectory were used to calculate LIE. Data represented here are averages of three independent MD simulation runs. (α = 018, β = 0.37 for CSN5i-3 and 0.45 for capzimin). Bold values refer to protein-protein complexes*.

We also investigated the binding of CSN5i-3, which is a very weak inhibitor of Rpn11. The starting structures of CSN5i-3 bound to the monomeric Rpn11 and Rpn8-Rpn11 heterodimer were generated by manual docking. The CSN5i-3-Rpn11 complex showed a very high Cα-RMSD (Figure 7A) suggesting that Rpn11 undergoes large conformational changes (Figure 9B). However, in the presence of Rpn8, the Cα-RMSD of Rpn11 was comparatively low (Figure 7A). The Ins-1 loop was more flexible in the absence of Rpn8 (Figure 7B). In CSN5i-3-Rpn11 complex, we observed that CSN5i-3 was stable (Figure 7C). The N-Zn^2+^ distance was very close to the distance reported in the crystal structure of CSN5 crystallized with CSN5i-3 (Figure 7D and Table 1). This simulated binding of CSN5i-3 to Rpn11 would not be in agreement with experiment and explain its low inhibitor activity. Thus, we investigated whether this was an unrealistic over binding, a protein-protein complex environment would be able to reproduce the selectivity.

In the heterodimeric Rpn8-Rpn11 complex, CSN5i-3 binding in the pocket was not stable (Figure 7C), the distance to zinc increases continously and eventually the ligand lost coordination with Zn^+2^ (Figure 7D and Table 1) which shows that the influence of Rpn8 on the capzimin bound Rpn11 is prominent. Interaction of CSN5i-3 in monomeric Rpn11 is shown in Figure 8A. In both complexes, H-bond with Thr129 was absent (Figure 8 and Figures S4C,D). We see that binding of CSN5i-3 to Rpn11 is significantly influenced by the presence of Rpn8 (Figures 7C,D, 8B) and the consideration of this heterodimeric state off Rpn11-Rpn8 is necessary to explain capzimin binding and CSN5i-3 non-binding. LIE calculations (Table 2) revealed that CSN5i-3 binds moderately to the monomeric Rpn11 and very weakly to the Rpn8-Rpn11 heterodimer. Outside the proteasome, Rpn11 also plays an important role in different cellular activities.^33^ Therefore, binding of capzimin and CSN5i-3 to the monomeric Rpn11 are physiologically possible. In recent study, CSN5i-3 has been shown to bind with the recombinant monomeric CSN5 and a co-crystallized structure has been obtained (Schlierf et al., 2016).

### Binding of capzimin and CSN5i-3 to monomeric CSN5 and CSN5-CSN6 heterodimer

**Figure 10:**
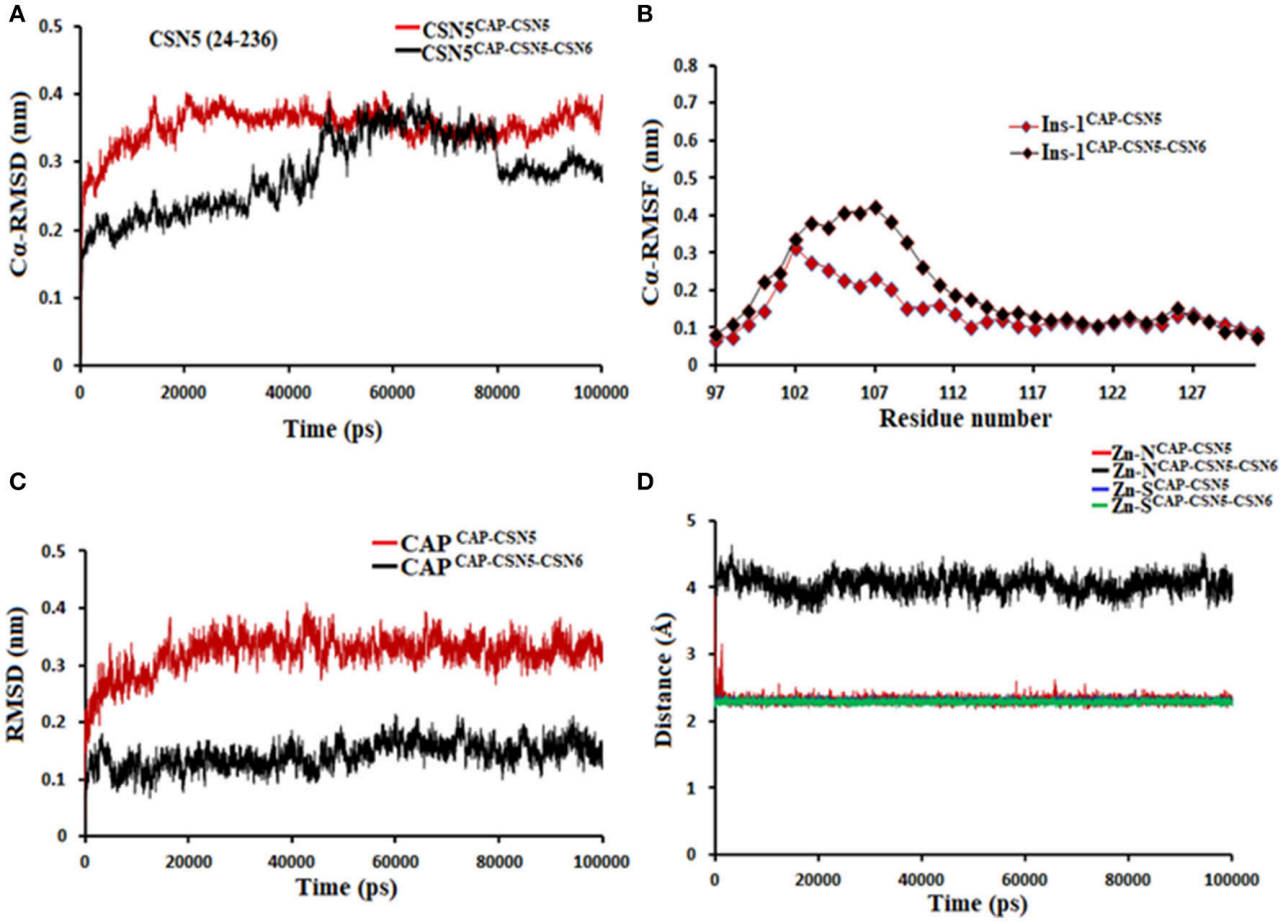
MD simulation data of capzimin bound to CSN5 in the absence (red) and presence of CSN6 (black). **(A)** Cα-RMSD of CSN5 **(B)** Cα-RMSF of Ins-1 region of CSN5 **(C)** RMSD of capzimin and **(D)** distances between coordinating atoms of capzimin and Zn^+2^. Zn^+2^-S distances in capzimin bound CSN5 and CSN5-CSN6 heterodimer are shown in blue and green color, respectively.

**Figure 11:**
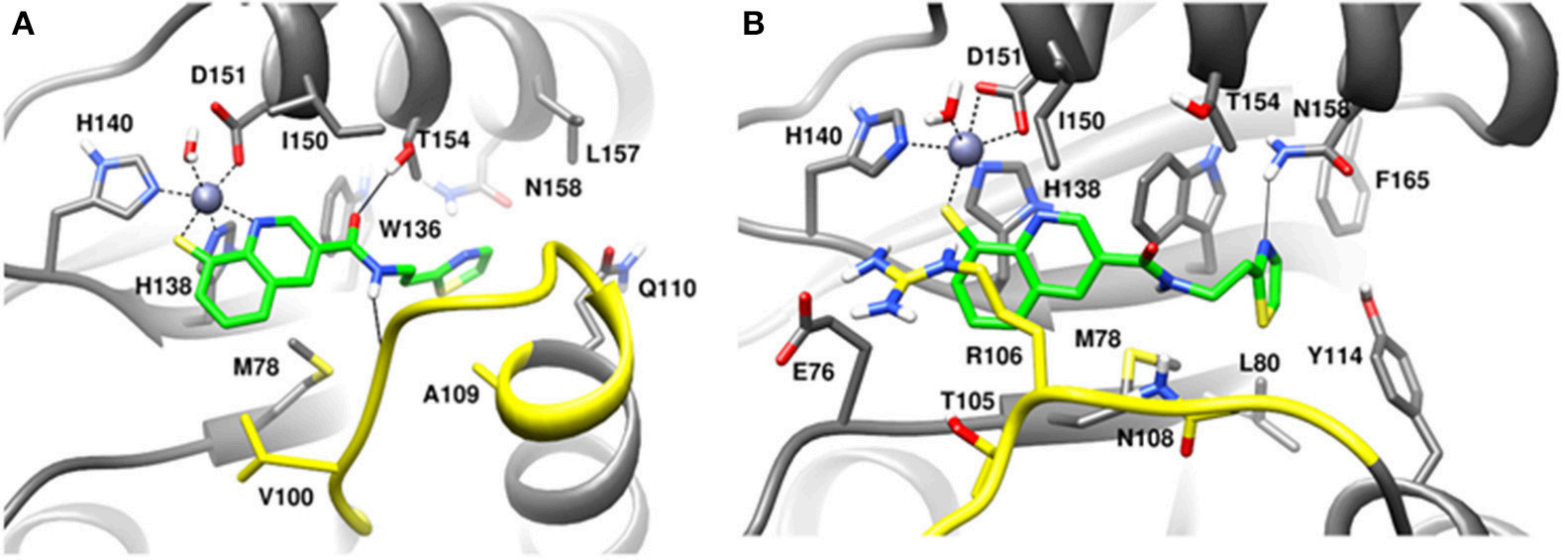
MD snapshots showing interactions of **(A)** capzimin with the CSN5 monomer and **(B)** capzimin with CSN5 in the presence of CSN6. Yellow region corresponds to Ins-1 loop. H-bonds are represented by solid black line.

MD simulation of capzimin with monomeric CSN5 and CSN5-CSN6 heterodimer revealed that its binding to CSN5 is also influenced by the CSN6 (Figure 10). In the presence of CSN6, the Ins-1 loop showed a larger flexibility (Figure 10B). Capzimin shows a higher ligand RMSD in the monomeric CSN5 (Figure 10C). In the monomeric CSN5, capzimin shows bidentate interactions with the catalytic zinc (Figures 10D, 11A) and stable H-bond between amide NH and Thr154 (Figure 11A and Figure S5A). Intermittent H-bonds with Glu101 and Tyr143 were also observed. However, in the presence of CSN6, capzimin only showed a mono-dentate coordination with catalytic zinc (Figures 10D, 11B) and low occupancy H-bond with Met78, Arg106, and Asn158 (Figure S5B). Thiazole and amide moieties of capzimin showed H-bonding with the side chains of Asn158 and Met78, respectively. The side chain of Arg106 of Ins-1 loop showed H-bond with the 8TQ fragment of capzimin. Apart from H-bond, the thiazole moiety also showed hydrophobic interactions with side chains of Met78, Leu80 and Phe165. LIE calculations show that capzimin binds more strongly to the monomeric CSN5 (Table 2).

**Figure 12:**
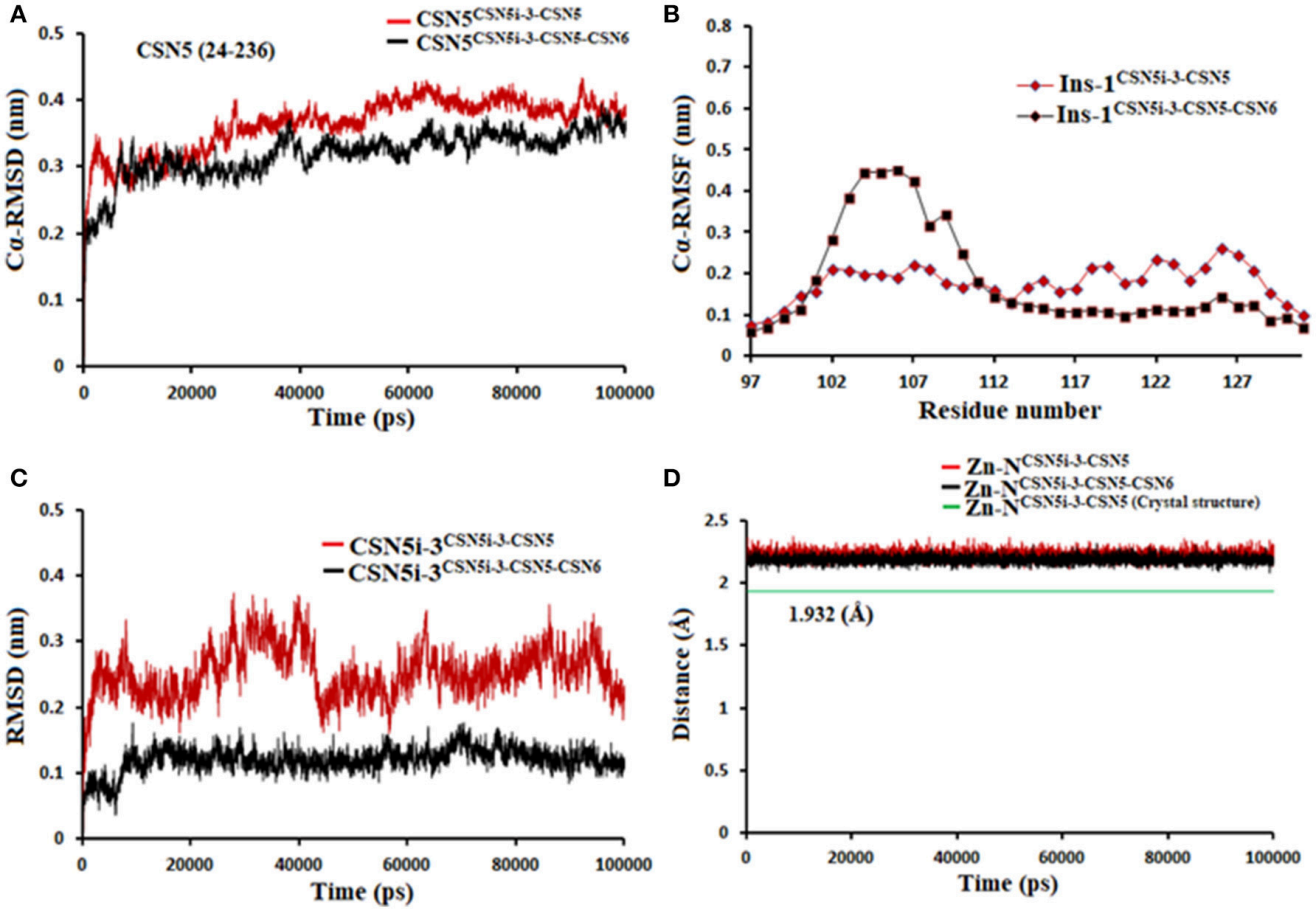
MD simulation data of CSN5i-3 bound to CSN5 in the absence (red) and presence of CSN6 (black). **(A)** Cα-RMSD of CSN5 **(B)** Cα-RMSF of Ins-1 region of CSN5 **(C)** RMSD of CSN5i-3 and **(D)** distances between coordinating atoms of CSN5i-3 and Zn^+2^.

**Figure 13:**
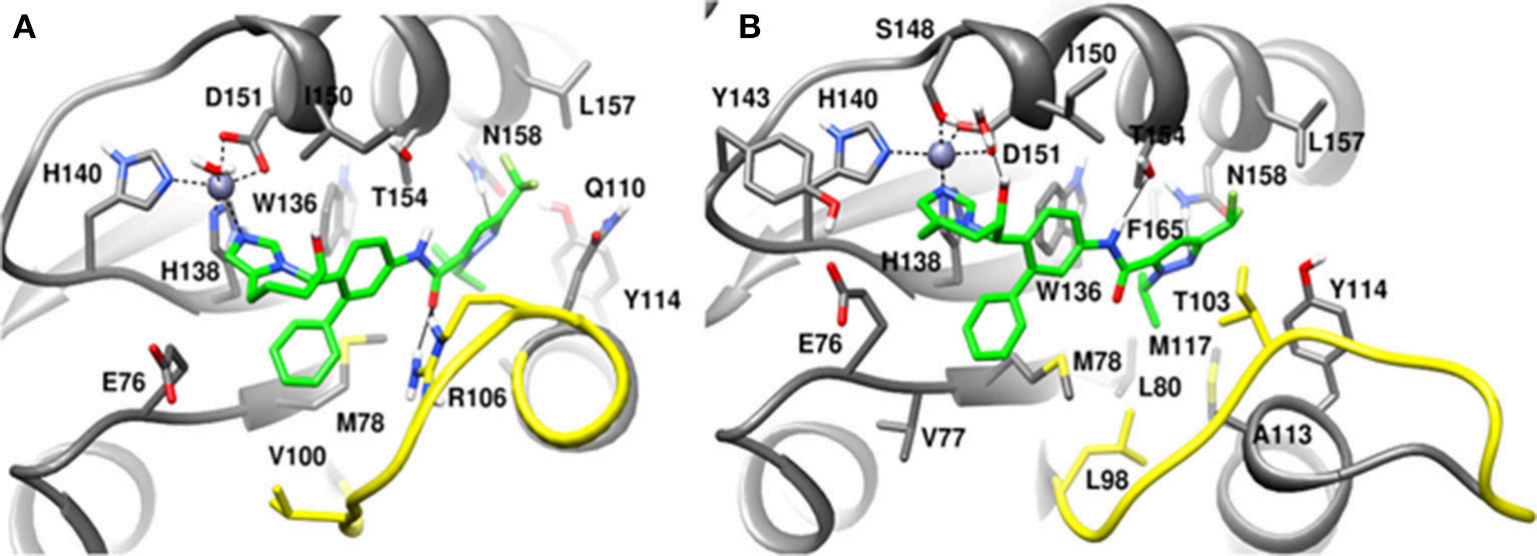
MD snapshots showing interaction of **(A)** CSN5i-3 with CSN5 monomer and **(B)** CSN5i-3 with CSN5 in the presence of CSN6. Yellow region corresponds to Ins-1 loop. H-bonds are represented by solid black line.

If we consider the crystal structure of CSN5 with CSN5i-3, a part of Ins-1 loop (100–106) is missing and residues of α4 helix do not interact with the CSN5i-3 ligand. The orientation of the α4 helix suggests that residues in the Ins-1 loop will also not make any interaction with the CSN5i-3. The MD simulation results (Figure 12) show that the Cα-RMSDs of CSN5 in both complexes do not vary much and they converge near the end of simulations (Figure 12A). In the presence of CSN6, Residues (101–110) in the Ins-1 loop (98–113) of CSN5 showed more flexibility but α4 helix (111–131) was comparatively less flexible (Figure 12B). Previous MD simulations study on monomeric CNS5 suggests that portions of the Ins-1 region show high flexibility (Echalier et al., 2013). In the CSN5-CSN6 heterodimer we observed that CSN5i-3 is stable (Figure 12C) and its conformation is very close to the conformation reported in the crystal structure (Figure S6). The N-Zn^2+^ distances are shown in Figure 12D. They are stable over time for the monomer and heterodimer states and only slightly longer than in the crystal structure. In both complexes, residues in Ins-1 loop make contact with the CSN5i-3 (Figure 13). We observed that interaction of the α2 helix of CSN6 affects the movement of the Ins-1 loop and α4 helix of CSN5.

**Figure 14:**
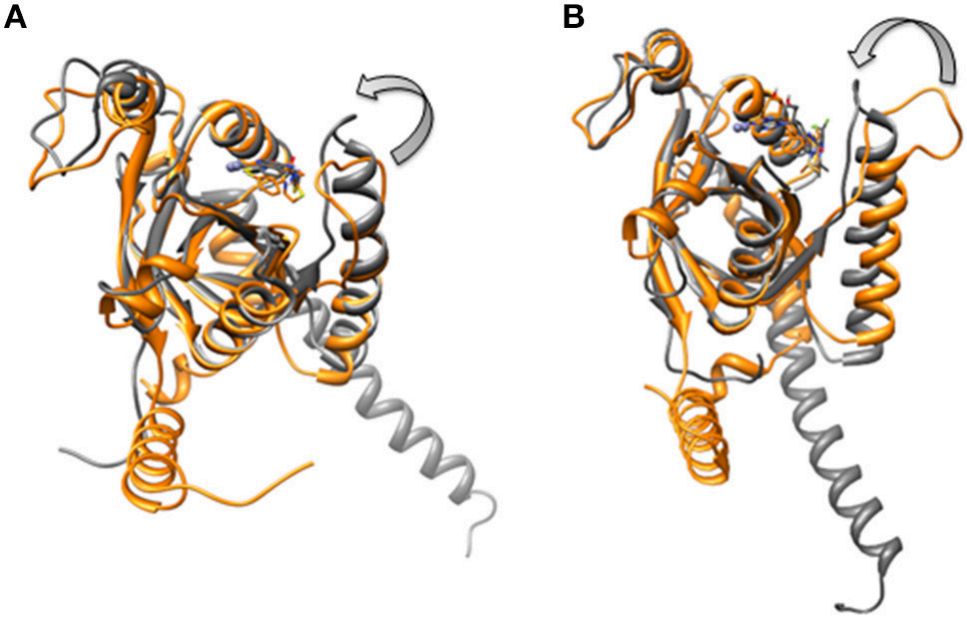
Aligned structures of inhibitors bound CSN5 in the absence (gray) and presence of CSN6 (orange) in which Ins-1 loop has moved (curved arrow) toward the distal ubiquitn binding site **(A)** capzimin bound CSN5 and **(B)** CSN5i-3 bound CSN5.

In the monomeric CSN5, CSN5i-3 makes a low occupancy H-bond with Thr154 and relatively stable H-bond with Asn158 (Figure 13A and Figure S5C). However, in the CSN5-CSN6 heterodimer, CSN5i-3 forms stable H-bonds with both Thr154 and Asn158 (Figure S5D and Figure 13B). In the crystal structure, the H-bond between the azole ring of CSN5i-3 and Asn158 is not present but our MD refinement showed a stable H-bond. The difluoromethyl group projects toward Leu157 and makes hydrophobic interactions with it (Figure 13). In both capzimin and CSN5i-3 bound monomeric CSN5, we observed that Ins-1 region slightly moves toward inhibitors and a part of Ins-1 loop changes into α helix (Figure 14). This may be the reason behind lower flexibility of Ins-1 loop in the monomeric CSN5 compared to the CSN6 bound CSN5.

### Structural elements responsible for ligand-selective inhibition of Rpn11 and CSN5

Based on the MD simulation results of Rpn11 and CSN5 with capzimin and CSN5i-3, we can explain the selective inhibition of these two proteins. At the sequence level, the MPN domain of CSN5 and Rpn11 are moderately conserved. However, the Zn^2+^ binding residues are fully conserved. The distal ubiquitin binding region is large therefore we mainly focused on the residues which interact with capzimin and CSN5i-3. We found that Leu98, Val100, Thr105, Arg106, Gln110, Ala112, Ala113, Tyr114, Glu115, Tyr116, Met117, A119, Ile150, Leu157 and Asn158, and Phe161 (in CSN5) are substituted by Met75, Gln77, Val82, Ser83, Glu85, Val87, Asp88, Pro89, Val90, Phe91, Gln92, Lys94, Val125, Ser132, Phe133, and Leu136, respectively (in Rpn11). We observed that most of the residues in the Ins-1 loop region are not conserved. The Ins-1 region plays an important role in positioning of C-terminus of the distal ubiquitin for cleavage of iso-peptide bond (Worden et al., 2014). Hence Ins-1 region appears very promising for the design of selective JAMM inhibitors.

If we consider the calculated LIE of capzimin in the monomeric Rpn11 and CSN5 proteins, we see that capzimin has a slightly higher affinity for Rpn11 (1 kcal/mole more than CSN5). Selective binding is more pronounced and can be rationalized when we consider their respective binding partners, Rpn8 and CSN6. Similarly, selective binding of CSN5i-3 is more pronounced when we consider the heterodimeric states of Rpn11 and CSN5.

Thus, we need to consider the heterodimeric states of Rpn11 and CSN5 to explain the selectivity of capzimin and CSN5i-3. In the presence of binding partners, the Ins-1 regions of RPN11 and CSN5 show different flexibility which in turn affects the binding of capzimin and CSN5i-3.

Capzimin is 80-fold more selective toward Rpn11 and its thiazole moiety appears important for this selectivity. In the Rpn8-Rpn11 heterodimer, capzimin showed a bidentate coordination with Zn^2+^ and a stable H-bond with side chain of Thr129 (Figure S4B). However, in the CSN5-CSN6 heterodimer, capzimin displayed a monodentate coordination with Zn^2+^ and only low occupancy H-bonds with side chains of Met78, Arg106, and Asn158 (Figure S5B). The low affinity of capzimin for CSN5 can be attributed to the lack of an extra N-Zn^2+^ coordination and H-bond with Thr154. We observed that in the case of capzimin, the binding energies were overestimated. The major reason for this overestimation can be attributed to the presence of a net negative charge on the capzimin ligand. A previous study has also reported very negative binding energy values for negatively charged ligands (Genheden and Ryde, 2015). It should be noted that LIE calculations are very sensitive to the β parameter. Values of β depend on the chemical nature of ligands and can have different values for different ligands. In present study, we have used LIE data only to compare relative binding affinities between monomeric and heterodimeric protein structures.

As for selective binding of CSN5i-3 to CSN5, we observed that in the presence of CSN6, CSN5i-3 binds strongly to the protein-protein complex. However in the case of the Rpn8-Rpn11 heterodimer, CSN5i-3 showed only very weak interaction. With the progress of the MD simulations, CSN5i-3 loses coordination with Zn^2+^ and H-bond with Thr129. In Rpn11, the side chain of Phe133 cannot form H-bond with the azole ring of CSN5i-3. Instead, the azole ring showed hydrophobic interaction with side chain of Leu56, Met75 and Phe133 and van der Waals interactions with the sidechain of Asp88.

It seems that establishing interactions of the azole ring with nearby sidechains resulted in loss of coordination with the active site Zn^2+^ ion. In the case of the Rpn8-Rpn11 heterodimer, LIE calculations showed only a very low binding energy of CSN5i-3 (Table 2) which is in agreement with the reported ligand binding selectivity. Overall our findings suggest that the heterodimeric protein states of both CSN5 and Rpn11 targets have to be considered to explain the selectivity of capzimin and CSN5i-3 ligands.

## Conclusions

In the present study the computational analysis of binding modes and selectivities of reported metallo-deubiquitinylase inhibitors, capzimin and CSN5i-3, was performed in detail. Capzimin is a selective inhibitor of Rpn11 while CSN5i-3 is selective for CSN5. We found that capzimin binds to RPN11 via chelation of the active site Zn^2+^ and its interaction extends to the distal ubiquitin binding site. Our MD studies suggest that compared to the monomeric states, the heterodimeric protein-protein complexes of Rpn11 and CSN5 are conformationally stable. Considering the Rpn8-Rpn11 and CSN5-CSN6 heterodimers, we found that residues in the distal ubiquitin site are responsible for selectivity and must be taken into consideration for the design of selective inhibitors of CSN5 and Rpn11 in future studies. Additionally, we have shown that flexibility of Ins-1 region in Rpn11 and CSN5 is significantly affected by the presence of their respective protein binding partners.

## Author contributions

MS and MN designed the study. VK performed simulations in this study. MS, MN, and VK analyzed and discussed the results and wrote the manuscript.

### Conflict of interest statement

The authors declare that the research was conducted in the absence of any commercial or financial relationships that could be construed as a potential conflict of interest.
