# Supplementary material for: Computational Studies on the Inhibitor Selectivity of Human JAMM Deubiquitinylases Rpn11 and CSN5
Source: Front Chem. 2018 Oct 9;6:480. doi: 10.3389/fchem.2018.00480 (PMC6189316; doi:10.3389/fchem.2018.00480)
Supplement: Supplementary file 1 [file Data_Sheet_1.PDF]

Supplementary information

Computational studies on the inhibitor selectivity of human JAMM deubiquitinylases Rpn11 and CSN5

Vikash Kumar[ǂ,‡] Michael Naumann[ǂ], Matthias Stein[ǂ,‡*]

[ǂ]Institute of Experimental and Internal Medicine, Medical Faculty, Otto von Guericke University, Leipziger Strasse 44, 39120 Magdeburg, Germany

[‡]Molecular Simulations and Design Group, Max Planck Institute for Dynamics of Complex Technical Systems, Sandtorstrasse 1, 39106 Magdeburg, Germany

*Corresponding author. Dr Matthias Stein, email matthias.stein@mpi-magdeburg.mpg.de

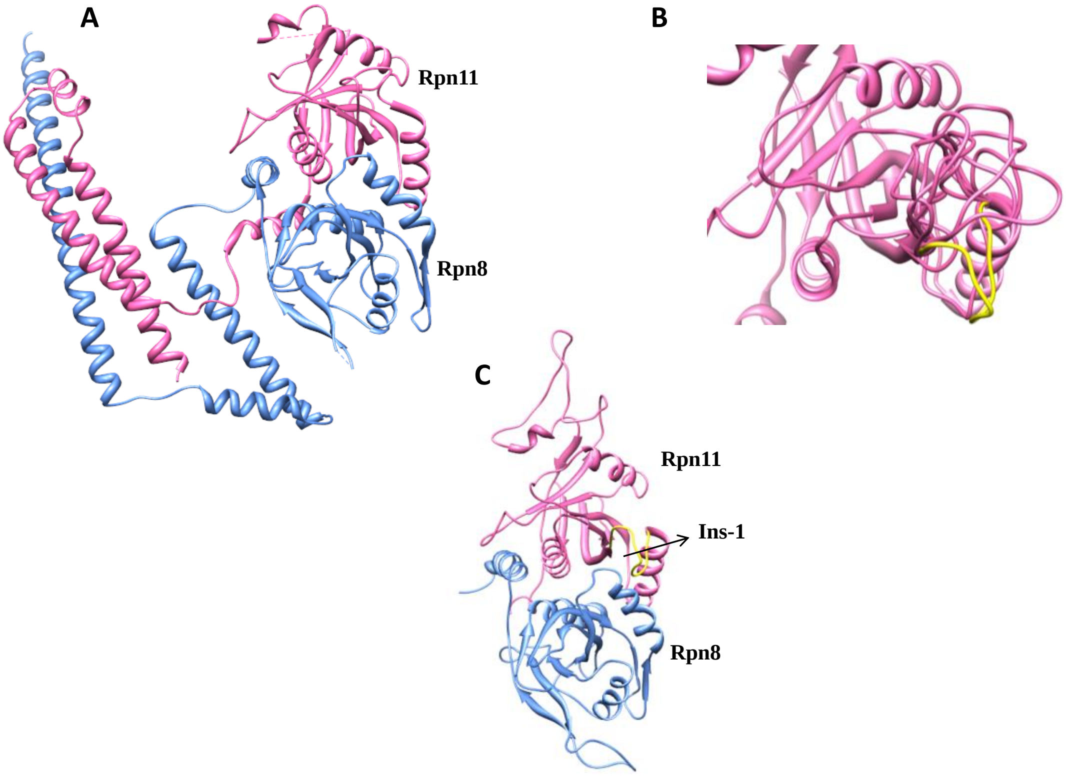

Figure. S1 (A) Unprocessed Rpn8-Rpn11 heterodimer (B) Ins-1 loop conformations in RPN11 generated by MODELLER and (C) processed Rpn8-Rpn11 heterodimer. RPN11 model having conformation of loop away from the catalytic site (highlighted in yellow color) was selected for further studies.

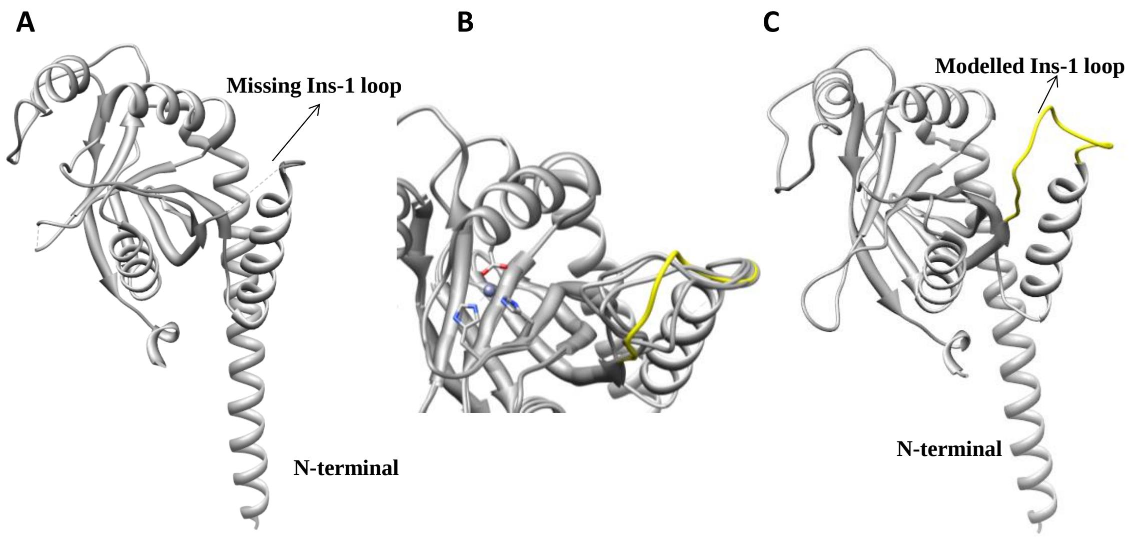

Figure S2 (A) Crystal structure of CSN5 with missing Ins-1 loop region (100-106) and (B) Ins-1 loop conformations in CSN5 generated by MODELLER and (C) processed structure of CSN5 in which Ins-1 loop has been modelled. CSN5 structure having conformation of loop away from the catalytic site (highlighted in yellow color) was selected for further studies.

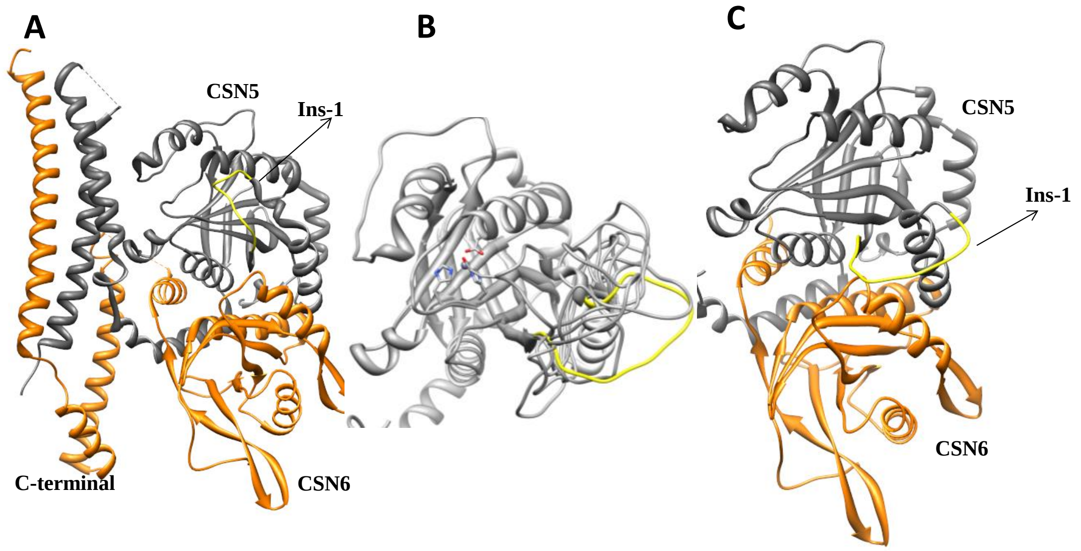

Figure S3 (A) Structure of CSN5-CSN6 extracted from the human COP9 signalosome in which Ins-1 loop (98-109) of CSN5 has obstructed the distal ubiquitin binding site (B) Ins-1 loop conformations in CSN5 generated by MODELLER and (C) processed structure of CSN5-CSN6 heterodimer in which Ins-1 loop has been modelled. CSN5 structure having conformation of loop away from the catalytic site (highlighted in yellow color) was selected for further studies.

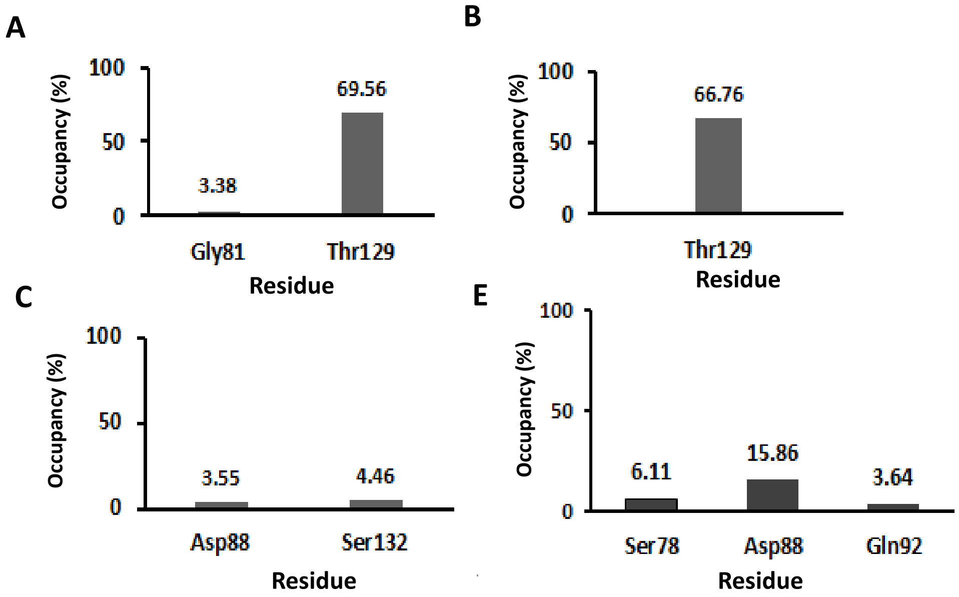

Figure S4 Occupancy of H-bond(s) during MD simlation. **(**A) Capzimin bound to isolated RPN11 (B) capzimin bound to RPN8-RPN11 heterodimer (C) CSN5i-3 bound to isolated RPN11 and (D) CSN5i-3 bound to RPN8-RPN11 heterodimer. Trajectories from all three independent runs were merged.

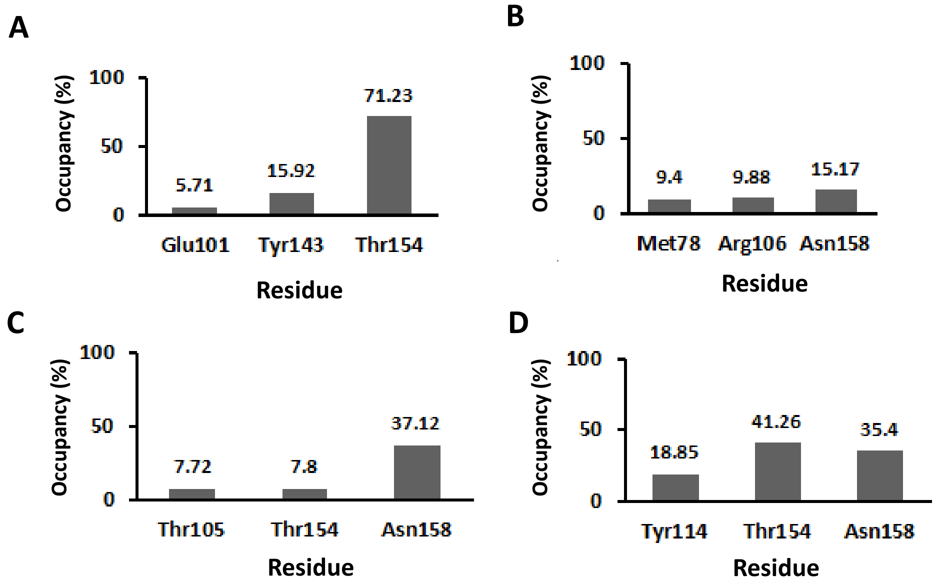

Figure S5 Occupancy of H-bond(s) during MD simlation. **(**A) Capzimin bound to isolated CSN5 (B) capzimin bound to CSN5-CSN6 heterodimer (C) CSN5i-3 bound to isolated CSN5 and (D) CSN5i-3 bound to CSN5-CSN6 heterodimer. Trajectories from all three independent runs were merged .

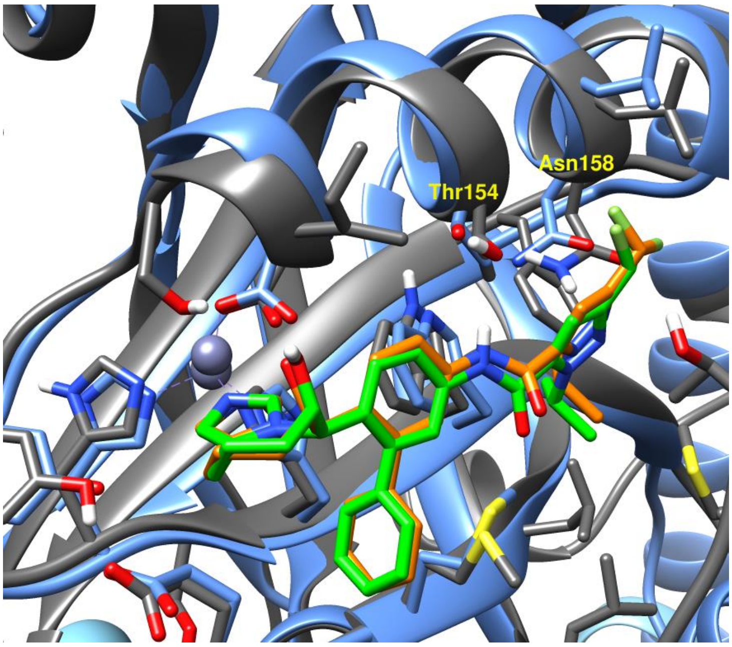

Figure S6 Overlay of X ray pose of CSN5i-3 (orange) with the representative snapshot from MD run (green). Crystal structure of CSN5 and snapshot from MD run are shown in hotpink and dim gray color respectively. Two residues Thr154 and Asn158 have been highlighted.

Table S1: Information regarding the systems used for the MD simulation studies

System	Box dimension (x, y , z)	Total atoms
CSN5i-3-CSN5	9.37, 7.36, 8.03	53730
CSN5i-3-CSN5-CSN6	8.13, 10.24, 7.94	65506
CAP-CSN5	9.36, 7.34, 8.01	53428
CAP-CSN5-CSN6	8.12, 10.23, 7.94	65561
CSN5i-3-RPN11	7.20, 7.65, 5.99	32262
CSN5i-3-RPN8-RPN11	7.70, 10.43, 6.91	54985
CAP-RPN11	7.18, 7.65, 6.01	32063
CAP-RPN8-RPN11	7.69, 10.43, 6.92	54818

Parameters of Capzimin and CSN5i-3:

Capzimin

; Built itp for CAP_DOCKED.mol2

; by user vzoete Mon Aug 7 19:48:52 CEST 2017

[atomtypes]

; name at.num mass charge ptype sigma epsilon

CB 6 12.0110 0.0 A 0.355005 0.292880

NPYD 7 14.0067 0.0 A 0.329632 0.836800

C5A 6 12.0110 0.0 A 0.363487 0.209200

C5B 6 12.0110 0.0 A 0.363487 0.209200

N5B 7 14.0067 0.0 A 0.329632 0.836800

S-P 16 32.0660 0.0 A 0.356359 1.882800

C=O 6 12.0110 0.0 A 0.356359 0.460240

O=C 8 15.9994 0.0 A 0.302905 0.502080

NC=O 7 14.0067 0.0 A 0.329632 0.836800

HNCO 1 1.0079 0.0 A 0.040001 0.192464

CR 6 12.0110 0.0 A 0.387541 0.230120

STHI 16 32.0660 0.0 A 0.356359 1.882800

HCMM 1 1.0079 0.0 A 0.235197 0.092048

[pairtypes]

; i j func sigma1-4 epsilon1-4 ; THESE ARE 1-4 INTERACTIONS

O=C CB 1 0.302228 0.383470

O=C NPYD 1 0.289542 0.648182

O=C C5A 1 0.306469 0.324091

O=C C5B 1 0.306469 0.324091

O=C N5B 1 0.289542 0.648182

O=C S-P 1 0.302905 0.972274

O=C C=O 1 0.302905 0.480705

O=C O=C 1 0.249452 0.502080

O=C NC=O 1 0.289542 0.648182

O=C HNCO 1 0.144726 0.310857

O=C CR 1 0.293997 0.144938

O=C STHI 1 0.302905 0.972274

O=C HCMM 1 0.242324 0.214978

CR CB 1 0.346773 0.110698

CR NPYD 1 0.334087 0.187114

CR C5A 1 0.351014 0.093557

CR C5B 1 0.351014 0.093557

CR N5B 1 0.334087 0.187114

CR S-P 1 0.347450 0.280671

CR C=O 1 0.347450 0.138768

CR NC=O 1 0.334087 0.187114

CR HNCO 1 0.189271 0.089737

CR CR 1 0.338541 0.041840

CR STHI 1 0.347450 0.280671

CR HCMM 1 0.286869 0.062059

[moleculetype]
; Name nrexcl
LIG 3
[atoms]
; nr type resnr resid atom cgnr charge mass
1 CB 1 LIG C1 1 -0.1500 12.0110
2 CB 1 LIG C2 2 -0.1500 12.0110
3 CB 1 LIG C3 3 -0.1500 12.0110

4 CB 1 LIG C4 4 -0.0470 12.0110
5 CB 1 LIG C5 5 0.3100 12.0110
6 CB 1 LIG C6 6 0.0000 12.0110
7 NPYD 1 LIG N1 7 -0.6200 14.0067
8 CB 1 LIG C7 8 0.1600 12.0110
9 CB 1 LIG C8 9 0.0862 12.0110
10 CB 1 LIG C9 10 -0.1500 12.0110
11 C5A 1 LIG C13 11 0.1981 12.0110
12 C5A 1 LIG C15 12 -0.1100 12.0110
13 C5B 1 LIG C14 13 0.0772 12.0110
14 N5B 1 LIG N3 14 -0.5653 14.0067
15 S-P 1 LIG S1 15 -0.9530 32.0660
16 C=O 1 LIG C10 16 0.5438 12.0110
17 O=C 1 LIG O1 17 -0.5700 15.9994
18 NC=O 1 LIG N2 18 -0.7301 14.0067
19 HNCO 1 LIG H7 19 0.3700 1.0079
20 CR 1 LIG C11 20 0.3001 12.0110
21 CR 1 LIG C12 21 0.1800 12.0110
22 STHI 1 LIG S2 22 -0.0800 32.0660
23 HCMM 1 LIG HC 23 0.1500 1.0079
24 HCMM 1 LIG HC1 24 0.1500 1.0079
25 HCMM 1 LIG HC2 25 0.1500 1.0079
26 HCMM 1 LIG HC3 26 0.1500 1.0079
27 HCMM 1 LIG HC4 27 0.1500 1.0079
28 HCMM 1 LIG HC5 28 0.0000 1.0079
29 HCMM 1 LIG HC6 29 0.0000 1.0079
30 HCMM 1 LIG HC7 30 0.0000 1.0079
31 HCMM 1 LIG HC8 31 0.0000 1.0079
32 HCMM 1 LIG HC9 32 0.1500 1.0079
33 HCMM 1 LIG HC10 33 0.1500 1.0079

[bonds]
; ai aj fu b0 kb, b0 kb
1 2 1 0.13740 335613.7 0.13740 335613.7
1 6 1 0.13740 335613.7 0.13740 335613.7
2 3 1 0.13740 335613.7 0.13740 335613.7
3 4 1 0.13740 335613.7 0.13740 335613.7
4 5 1 0.13740 335613.7 0.13740 335613.7
4 15 1 0.15000 252929.5 0.15000 252929.5
5 6 1 0.13740 335613.7 0.13740 335613.7
5 7 1 0.13330 345489.6 0.13330 345489.6
6 10 1 0.13740 335613.7 0.13740 335613.7
7 8 1 0.13330 345489.6 0.13330 345489.6
8 9 1 0.13740 335613.7 0.13740 335613.7
9 10 1 0.13740 335613.7 0.13740 335613.7
9 16 1 0.14570 270273.8 0.14570 270273.8
16 17 1 0.12220 779866.6 0.12220 779866.6
16 18 1 0.13690 351030.1 0.13690 351030.1
18 19 1 0.10150 401254.8 0.10150 401254.8
18 20 1 0.14360 280872.8 0.14360 280872.8

```
20  21 1 0.15080  256422.3  0.15080  256422.3
21  11 1 0.14710  269852.1  0.14710  269852.1
11  22 1 0.17170  216134.6  0.17170  216134.6
11  14 1 0.13130  501403.0  0.13130  501403.0
22  12 1 0.17170  216134.6  0.17170  216134.6
12  13 1 0.13770  428655.8  0.13770  428655.8
13  14 1 0.13690  268346.7  0.13690  268346.7
23   1 1 0.10840  319534.6  0.10840  319534.6
24   2 1 0.10840  319534.6  0.10840  319534.6
25   3 1 0.10840  319534.6  0.10840  319534.6
26   8 1 0.10840  319534.6  0.10840  319534.6
27  10 1 0.10840  319534.6  0.10840  319534.6
28  20 1 0.10930  287014.9  0.10930  287014.9
29  20 1 0.10930  287014.9  0.10930  287014.9
30  21 1 0.10930  287014.9  0.10930  287014.9
31  21 1 0.10930  287014.9  0.10930  287014.9
32  12 1 0.10800  333084.1  0.10800  333084.1
33  13 1 0.10800  331578.7  0.10800  331578.7

[ pairs ]
; ai aj fu
1   4 1
1  25 1
1   7 1
1   9 1
1  27 1
2   5 1
2  10 1
2  15 1
3   6 1
3  23 1
3   7 1
4  24 1
4  10 1
4   8 1
5  25 1
5  23 1
5   9 1
5  27 1
5  26 1
6  24 1
6  15 1
6   8 1
6  16 1
7  15 1
7  10 1
7  16 1
8  27 1
8  17 1
8  18 1
```

9 19 1
9 20 1
10 23 1
10 26 1
10 17 1
10 18 1
11 33 1
11 18 1
11 28 1
11 29 1
11 32 1
12 21 1
13 21 1
14 20 1
14 30 1
14 31 1
14 32 1
15 25 1
16 26 1
16 27 1
16 21 1
16 28 1
16 29 1
17 19 1
17 20 1
18 30 1
18 31 1
19 21 1
19 28 1
19 29 1
20 22 1
22 30 1
22 31 1
22 33 1
23 24 1
24 25 1
28 30 1
28 31 1
29 30 1
29 31 1
32 33 1

[angles]
; ai aj ak fu th0 kth ub0 kub th0 kth ub0 kub
2   1   6 1 119.9770 402.88   119.9770 402.88
2   1 23 1 120.5710 339.05   120.5710 339.05
6   1 23 1 120.5710 339.05   120.5710 339.05
1   2   3 1 119.9770 402.88   119.9770 402.88
1   2 24 1 120.5710 339.05   120.5710 339.05
3   2 24 1 120.5710 339.05   120.5710 339.05

**2**	**3**	**4**	**1**	**119.9770**	**402.88**	**119.9770**	**402.88**
**2**	**3**	**25**	**1**	**120.5710**	**339.05**	**120.5710**	**339.05**
**4**	**3**	**25**	**1**	**120.5710**	**339.05**	**120.5710**	**339.05**
**3**	**4**	**5**	**1**	**119.9770**	**402.88**	**119.9770**	**402.88**
**3**	**4**	**15**	**1**	**120.5710**	**339.05**	**120.5710**	**339.05**
**5**	**4**	**15**	**1**	**120.5710**	**339.05**	**120.5710**	**339.05**
**4**	**5**	**6**	**1**	**119.9770**	**402.88**	**119.9770**	**402.88**
**4**	**5**	**7**	**1**	**126.1390**	**358.92**	**126.1390**	**358.92**
**6**	**5**	**7**	**1**	**126.1390**	**358.92**	**126.1390**	**358.92**
**1**	**6**	**5**	**1**	**119.9770**	**402.88**	**119.9770**	**402.88**
**1**	**6**	**10**	**1**	**119.9770**	**402.88**	**119.9770**	**402.88**
**5**	**6**	**10**	**1**	**119.9770**	**402.88**	**119.9770**	**402.88**
**5**	**7**	**8**	**1**	**115.4060**	**653.40**	**115.4060**	**653.40**
**7**	**8**	**9**	**1**	**126.1390**	**358.92**	**126.1390**	**358.92**
**7**	**8**	**26**	**1**	**115.5880**	**417.33**	**115.5880**	**417.33**
**9**	**8**	**26**	**1**	**120.5710**	**339.05**	**120.5710**	**339.05**
**8**	**9**	**10**	**1**	**119.9770**	**402.88**	**119.9770**	**402.88**
**8**	**9**	**16**	**1**	**114.4750**	**480.57**	**114.4750**	**480.57**
**10**	**9**	**16**	**1**	**114.4750**	**480.57**	**114.4750**	**480.57**
**6**	**10**	**9**	**1**	**119.9770**	**402.88**	**119.9770**	**402.88**
**6**	**10**	**27**	**1**	**120.5710**	**339.05**	**120.5710**	**339.05**
**9**	**10**	**27**	**1**	**120.5710**	**339.05**	**120.5710**	**339.05**
**14**	**11**	**21**	**1**	**127.6100**	**520.91**	**127.6100**	**520.91**
**14**	**11**	**22**	**1**	**114.5160**	**514.29**	**114.5160**	**514.29**
**21**	**11**	**22**	**1**	**122.1010**	**543.19**	**122.1010**	**543.19**
**13**	**12**	**22**	**1**	**108.4800**	**513.69**	**108.4800**	**513.69**
**13**	**12**	**32**	**1**	**131.7210**	**347.47**	**131.7210**	**347.47**
**22**	**12**	**32**	**1**	**126.1410**	**236.67**	**126.1410**	**236.67**
**12**	**13**	**14**	**1**	**111.6210**	**625.09**	**111.6210**	**625.09**
**12**	**13**	**33**	**1**	**126.1700**	**301.71**	**126.1700**	**301.71**
**14**	**13**	**33**	**1**	**120.4780**	**420.94**	**120.4780**	**420.94**
**11**	**14**	**13**	**1**	**103.7790**	**726.27**	**103.7790**	**726.27**
**9**	**16**	**17**	**1**	**119.9680**	**442.02**	**119.9680**	**442.02**
**9**	**16**	**18**	**1**	**112.4950**	**663.03**	**112.4950**	**663.03**
**17**	**16**	**18**	**1**	**127.1520**	**546.20**	**127.1520**	**546.20**
**16**	**18**	**19**	**1**	**120.2770**	**346.27**	**120.2770**	**346.27**
**16**	**18**	**20**	**1**	**119.6000**	**494.41**	**119.6000**	**494.41**
**19**	**18**	**20**	**1**	**120.0660**	**332.42**	**120.0660**	**332.42**
**18**	**20**	**21**	**1**	**109.9600**	**632.32**	**109.9600**	**632.32**
**18**	**20**	**28**	**1**	**107.6460**	**445.64**	**107.6460**	**445.64**
**18**	**20**	**29**	**1**	**107.6460**	**445.64**	**107.6460**	**445.64**
**21**	**20**	**28**	**1**	**110.5490**	**383.00**	**110.5490**	**383.00**
**21**	**20**	**29**	**1**	**110.5490**	**383.00**	**110.5490**	**383.00**
**28**	**20**	**29**	**1**	**108.8360**	**310.74**	**108.8360**	**310.74**
**11**	**21**	**20**	**1**	**110.0580**	**605.82**	**110.0580**	**605.82**
**11**	**21**	**30**	**1**	**110.4670**	**373.97**	**110.4670**	**373.97**
**11**	**21**	**31**	**1**	**110.4670**	**373.97**	**110.4670**	**373.97**
**20**	**21**	**30**	**1**	**110.5490**	**383.00**	**110.5490**	**383.00**
**20**	**21**	**31**	**1**	**110.5490**	**383.00**	**110.5490**	**383.00**
**30**	**21**	**31**	**1**	**108.8360**	**310.74**	**108.8360**	**310.74**

```
11  22  12 1   88.4950 1181.54     88.4950 1181.54

[ dihedrals ]
; ai aj ak al fu phi0 kphi mult phi0 kphi mult
1   2   3   4 9 180.00  14.6440 2   180.00  14.6440 2
1   2   3  25 9 180.00  14.6440 2   180.00  14.6440 2
1   6   5   4 9 180.00  14.6440 2   180.00  14.6440 2
1   6   5   7 9 180.00  14.6440 2   180.00  14.6440 2
1   6  10   9 9 180.00  14.6440 2   180.00  14.6440 2
1   6  10  27 9 180.00  14.6440 2   180.00  14.6440 2
2   1   6   5 9 180.00  14.6440 2   180.00  14.6440 2
2   1   6  10 9 180.00  14.6440 2   180.00  14.6440 2
2   3   4   5 9 180.00  14.6440 2   180.00  14.6440 2
2   3   4  15 9 180.00  14.6440 2   180.00  14.6440 2
3   2   1   6 9 180.00  14.6440 2   180.00  14.6440 2
3   2   1  23 9 180.00  14.6440 2   180.00  14.6440 2
3   4   5   6 9 180.00  14.6440 2   180.00  14.6440 2
3   4   5   7 9 180.00  14.6440 2   180.00  14.6440 2
4   3   2  24 9 180.00  14.6440 2   180.00  14.6440 2
4   5   6  10 9 180.00  14.6440 2   180.00  14.6440 2
4   5   7   8 9 180.00  14.6440 2   180.00  14.6440 2
5   4   3  25 9 180.00  14.6440 2   180.00  14.6440 2
5   6   1  23 9 180.00  14.6440 2   180.00  14.6440 2
5   6  10   9 9 180.00  14.6440 2   180.00  14.6440 2
5   6  10  27 9 180.00  14.6440 2   180.00  14.6440 2
5   7   8   9 9 180.00  14.6440 2   180.00  14.6440 2
5   7   8  26 9 180.00  14.6440 2   180.00  14.6440 2
6   1   2  24 9 180.00  14.6440 2   180.00  14.6440 2
6   5   4  15 9 180.00  14.6440 2   180.00  14.6440 2
6   5   7   8 9 180.00  14.6440 2   180.00  14.6440 2
6  10   9   8 9 180.00  14.6440 2   180.00  14.6440 2
6  10   9  16 9 180.00  14.6440 2   180.00  14.6440 2
7   5   4  15 9 180.00  14.6440 2   180.00  14.6440 2
7   5   6  10 9 180.00  14.6440 2   180.00  14.6440 2
7   8   9  10 9 180.00  14.6440 2   180.00  14.6440 2
7   8   9  16 9 180.00  14.6440 2   180.00  14.6440 2
8   9  10  27 9 180.00  14.6440 2   180.00  14.6440 2
8   9  16  17 9 180.00   4.7196 2   180.00   4.7196 2
8   9  16  18 9 180.00   5.2300 2   180.00   5.2300 2
9  16  18  19 9 180.00  12.5520 2   180.00  12.5520 2
9  16  18  20 9 180.00  12.5520 2   180.00  12.5520 2
10   6   1  23 9 180.00  14.6440 2   180.00  14.6440 2
10   9   8  26 9 180.00  14.6440 2   180.00  14.6440 2
10   9  16  17 9 180.00   4.7196 2   180.00   4.7196 2
10   9  16  18 9 180.00   5.2300 2   180.00   5.2300 2
11  14  13  12 9 180.00  14.6440 2   180.00  14.6440 2
11  14  13  33 9 180.00  14.6440 2   180.00  14.6440 2
11  21  20  18 9   0.00   0.6276 3     0.00   0.6276 3
11  21  20  28 9   0.00   0.6276 3     0.00   0.6276 3
11  21  20  29 9   0.00   0.6276 3     0.00   0.6276 3
```

11	22	12	13	9	180.00	14.6440	2	180.00	14.6440	2
11	22	12	32	9	180.00	14.6440	2	180.00	14.6440	2
12	22	11	14	9	180.00	14.6440	2	180.00	14.6440	2
12	22	11	21	9	180.00	14.6440	2	180.00	14.6440	2
13	14	11	21	9	180.00	14.6440	2	180.00	14.6440	2
13	14	11	22	9	180.00	14.6440	2	180.00	14.6440	2
14	13	12	22	9	180.00	14.6440	2	180.00	14.6440	2
14	13	12	32	9	180.00	14.6440	2	180.00	14.6440	2
15	4	3	25	9	180.00	14.6440	2	180.00	14.6440	2
16	9	8	26	9	180.00	4.1840	2	180.00	4.1840	2
16	9	10	27	9	180.00	4.1840	2	180.00	4.1840	2
16	18	20	21	9	0.00	-2.1464	1	0.00	-2.1464	1
16	18	20	21	9	180.00	1.4518	2	180.00	1.4518	2
16	18	20	21	9	0.00	1.9832	3	0.00	1.9832	3
16	18	20	28	9	0.00	-4.3932	1	0.00	-4.3932	1
16	18	20	28	9	180.00	2.8493	2	180.00	2.8493	2
16	18	20	28	9	0.00	0.0460	3	0.00	0.0460	3
16	18	20	29	9	0.00	-4.3932	1	0.00	-4.3932	1
16	18	20	29	9	180.00	2.8493	2	180.00	2.8493	2
16	18	20	29	9	0.00	0.0460	3	0.00	0.0460	3
17	16	18	19	9	0.00	3.0041	1	0.00	3.0041	1
17	16	18	19	9	180.00	10.4056	2	180.00	10.4056	2
17	16	18	19	9	0.00	-0.9498	3	0.00	-0.9498	3
17	16	18	20	9	0.00	-0.6694	1	0.00	-0.6694	1
17	16	18	20	9	180.00	13.1670	2	180.00	13.1670	2
17	16	18	20	9	0.00	-0.3054	3	0.00	-0.3054	3
18	20	21	30	9	0.00	0.8912	3	0.00	0.8912	3
18	20	21	31	9	0.00	0.8912	3	0.00	0.8912	3
19	18	20	21	9	0.00	1.1548	1	0.00	1.1548	1
19	18	20	21	9	180.00	-0.7950	2	180.00	-0.7950	2
19	18	20	21	9	0.00	0.6820	3	0.00	0.6820	3
19	18	20	28	9	0.00	-1.2887	1	0.00	-1.2887	1
19	18	20	28	9	0.00	0.5732	3	0.00	0.5732	3
19	18	20	29	9	0.00	-1.2887	1	0.00	-1.2887	1
19	18	20	29	9	0.00	0.5732	3	0.00	0.5732	3
22	12	13	33	9	180.00	14.6440	2	180.00	14.6440	2
23	1	2	24	9	180.00	14.6440	2	180.00	14.6440	2
24	2	3	25	9	180.00	14.6440	2	180.00	14.6440	2
28	20	21	30	9	0.00	0.5941	1	0.00	0.5941	1
28	20	21	30	9	180.00	-2.8995	2	180.00	-2.8995	2
28	20	21	30	9	0.00	0.6569	3	0.00	0.6569	3
28	20	21	31	9	0.00	0.5941	1	0.00	0.5941	1
28	20	21	31	9	180.00	-2.8995	2	180.00	-2.8995	2
28	20	21	31	9	0.00	0.6569	3	0.00	0.6569	3
29	20	21	30	9	0.00	0.5941	1	0.00	0.5941	1
29	20	21	30	9	180.00	-2.8995	2	180.00	-2.8995	2
29	20	21	30	9	0.00	0.6569	3	0.00	0.6569	3
29	20	21	31	9	0.00	0.5941	1	0.00	0.5941	1
29	20	21	31	9	180.00	-2.8995	2	180.00	-2.8995	2
29	20	21	31	9	0.00	0.6569	3	0.00	0.6569	3

```
32  12  13  33 9 180.00  14.6440 2   180.00  14.6440 2

[ dihedrals ]
; ai aj ak al fu xi0 kxi xi0 kxi
1   2   6  23 2   0.00   9.0291     0.00   9.0291
2   3   1  24 2   0.00   9.0291     0.00   9.0291
3   4   2  25 2   0.00   9.0291     0.00   9.0291
4   5   3  15 2   0.00  21.0790     0.00  21.0790
5   6   4   7 2   0.00  21.0790     0.00  21.0790
8   9   7  26 2   0.00  27.6981     0.00  27.6981
6   5   1  10 2   0.00  21.0790     0.00  21.0790
9  10   8  16 2   0.00  16.2590     0.00  16.2590
16  18   9  17 2   0.00  78.2910     0.00  78.2910
18  20  16  19 2   0.00 -12.0416     0.00 -12.0416
20  21  18  28 2   0.00   0.0000     0.00   0.0000
20  21  18  29 2   0.00   0.0000     0.00   0.0000
21  11  20  30 2   0.00   0.0000     0.00   0.0000
21  11  20  31 2   0.00   0.0000     0.00   0.0000
11  22  21  14 2   0.00  30.1081     0.00  30.1081
12  13  22  32 2   0.00   8.4349     0.00   8.4349
10   9   6  27 2   0.00   9.0291     0.00   9.0291
13  14  12  33 2   0.00  25.8990     0.00  25.8990
```

CSN5i-3:

```
; ----
; Built itp for CSN.mol2
;    by user vzoete     Tue Feb 21 13:46:11 CET 2017
; ----
;

[ atomtypes ]
; name at.num  mass   charge  ptype    sigma          epsilon
C5A     6   12.0110  0.0  A        0.363487    0.209200
CB      6   12.0110  0.0  A        0.355005    0.292880
C5B     6   12.0110  0.0  A        0.363487    0.209200
NPYL    7   14.0067  0.0  A        0.306469    0.376560
N5B     7   14.0067  0.0  A        0.329632    0.836800
N5A     7   14.0067  0.0  A        0.329632    0.836800
CR      6   12.0110  0.0  A        0.387541    0.230120
C=O     6   12.0110  0.0  A        0.356359    0.460240
NC=O    7   14.0067  0.0  A        0.329632    0.836800
OR      8   15.9994  0.0  A        0.315378    0.636386
O=C     8   15.9994  0.0  A        0.302905    0.502080
F       9   18.9984  0.0  A        0.290433    0.564840
HCMM    1    1.0079  0.0  A        0.235197    0.092048
```

```
HNCO    1    1.0079  0.0  A        0.040001    0.192464
HOR     1    1.0079  0.0  A        0.040001    0.192464


[ pairtypes ]
;  i     j    func     sigma1-4       epsilon1-4 ; THESE ARE 1-4 INTERACTIONS
CR      C5A    1      0.351014    0.093557
CR      CB     1      0.346773    0.110698
CR      C5B    1      0.351014    0.093557
CR      NPYL   1      0.322505    0.125520
CR      N5B    1      0.334087    0.187114
CR      N5A    1      0.334087    0.187114
CR      CR     1      0.338541    0.041840
CR      C=O    1      0.347450    0.138768
CR      NC=O   1      0.334087    0.187114
CR      OR     1      0.326960    0.163176
CR      O=C    1      0.293997    0.144938
CR      F      1      0.314487    0.153730
CR      HCMM   1      0.286869    0.062059
CR      HNCO   1      0.189271    0.089737
CR      HOR    1      0.189271    0.089737
O=C     C5A    1      0.306469    0.324091
O=C     CB     1      0.302228    0.383470
O=C     C5B    1      0.306469    0.324091
O=C     NPYL   1      0.277960    0.434814
O=C     N5B    1      0.289542    0.648182
O=C     N5A    1      0.289542    0.648182
O=C     C=O    1      0.302905    0.480705
O=C     NC=O   1      0.289542    0.648182
O=C     OR     1      0.282415    0.565258
O=C     O=C    1      0.249452    0.502080
O=C     F      1      0.269942    0.532536
O=C     HCMM   1      0.242324    0.214978
O=C     HNCO   1      0.144726    0.310857
O=C     HOR    1      0.144726    0.310857

[ moleculetype ]
; Name nrexcl
LIG 3

[ atoms ]
; nr type resnr resid atom cgnr charge mass
   1 C5A  1  LIG C1      1 -0.3316  12.0110
   2 C5A  1  LIG C3      2  0.0365  12.0110
```

```
 3 CB   1  LIG C11     3 -0.1500  12.0110
 4 CB   1  LIG C12     4  0.0000  12.0110
 5 CB   1  LIG C14     5  0.0000  12.0110
 6 CB   1  LIG C20     6 -0.1500  12.0110
 7 CB   1  LIG C21     7 -0.1500  12.0110
 8 CB   1  LIG C22     8 -0.1500  12.0110
 9 CB   1  LIG C23     9 -0.1500  12.0110
10 CB   1  LIG C24    10 -0.1500  12.0110
11 C5A  1  LIG C26    11 -0.2366  12.0110
12 C5B  1  LIG C28    12 -0.1500  12.0110
13 C5B  1  LIG C29    13  0.1078  12.0110
14 NPYL 1  LIG N2     14  0.0476  14.0067
15 N5B  1  LIG N4     15 -0.5653  14.0067
16 C5B  1  LIG C5     16  0.0772  12.0110
17 CB   1  LIG C7     17 -0.1435  12.0110
18 CB   1  LIG C8     18 -0.1500  12.0110
19 CB   1  LIG C9     19 -0.1500  12.0110
20 CB   1  LIG C10    20  0.1170  12.0110
21 N5A  1  LIG N30    21 -0.7068  14.0067
22 NPYL 1  LIG N31    22  0.3140  14.0067
23 CR   1  LIG C15    23  0.1800  12.0110
24 CR   1  LIG C16    24  0.0000  12.0110
25 CR   1  LIG C17    25  0.0000  12.0110
26 CR   1  LIG C18    26  0.2800  12.0110
27 C=O  1  LIG C25    27  0.7150  12.0110
28 CR   1  LIG C6     28  0.3991  12.0110
29 NC=O 1  LIG N13    29 -0.5470  14.0067
30 OR   1  LIG O19    30 -0.6800  15.9994
31 O=C  1  LIG O27    31 -0.5700  15.9994
32 CR   1  LIG C32    32  0.2556  12.0110
33 CR   1  LIG C33    33  0.8610  12.0110
34 CR   1  LIG C34    34  0.0000  12.0110
35 CR   1  LIG C35    35  0.0000  12.0110
36 F    1  LIG F36    36 -0.3400  18.9984
37 F    1  LIG F37    37 -0.3400  18.9984
38 HCMM 1  LIG H3     38  0.1500   1.0079
39 HCMM 1  LIG H11    39  0.1500   1.0079
40 HCMM 1  LIG H152   40  0.0000   1.0079
41 HCMM 1  LIG H153   41  0.0000   1.0079
42 HCMM 1  LIG H162   42  0.0000   1.0079
43 HCMM 1  LIG H163   43  0.0000   1.0079
44 HCMM 1  LIG H172   44  0.0000   1.0079
45 HCMM 1  LIG H173   45  0.0000   1.0079
46 HCMM 1  LIG H18    46  0.0000   1.0079
```

```
  47 HCMM 1  LIG H20    47  0.1500   1.0079
  48 HCMM 1  LIG H21    48  0.1500   1.0079
  49 HCMM 1  LIG H22    49  0.1500   1.0079
  50 HCMM 1  LIG H23    50  0.1500   1.0079
  51 HCMM 1  LIG H24    51  0.1500   1.0079
  52 HCMM 1  LIG H28    52  0.1500   1.0079
  53 HCMM 1  LIG H5     53  0.1500   1.0079
  54 HCMM 1  LIG H6     54  0.0000   1.0079
  55 HCMM 1  LIG H8     55  0.1500   1.0079
  56 HCMM 1  LIG H9     56  0.1500   1.0079
  57 HNCO 1  LIG H13    57  0.3700   1.0079
  58 HCMM 1  LIG H32    58  0.0000   1.0079
  59 HCMM 1  LIG H33    59  0.0000   1.0079
  60 HCMM 1  LIG H351   60  0.0000   1.0079
  61 HCMM 1  LIG H352   61  0.0000   1.0079
  62 HCMM 1  LIG H353   62  0.0000   1.0079
  63 HOR  1  LIG H19    63  0.4000   1.0079
  64 HCMM 1  LIG H341   64  0.0000   1.0079
  65 HCMM 1  LIG H342   65  0.0000   1.0079
  66 HCMM 1  LIG H343   66  0.0000   1.0079

[ bonds ]
; ai aj fu b0 kb, b0 kb
  1  23 1 0.14710  269852.1  0.14710  269852.1
  1  14 1 0.13640  379454.5  0.13640  379454.5
  1  16 1 0.13770  428655.8  0.13770  428655.8
 20   3 1 0.13740  335613.7  0.13740  335613.7
 20  19 1 0.13740  335613.7  0.13740  335613.7
 20  29 1 0.13950  330133.5  0.13950  330133.5
  3   4 1 0.13740  335613.7  0.13740  335613.7
  4   5 1 0.13740  335613.7  0.13740  335613.7
  4  17 1 0.13740  335613.7  0.13740  335613.7
  5   6 1 0.13740  335613.7  0.13740  335613.7
  5  10 1 0.13740  335613.7  0.13740  335613.7
 23  24 1 0.15080  256422.3  0.15080  256422.3
 24  25 1 0.15080  256422.3  0.15080  256422.3
 25  26 1 0.15080  256422.3  0.15080  256422.3
 26  28 1 0.15080  256422.3  0.15080  256422.3
 26  30 1 0.14180  303937.5  0.14180  303937.5
  6   7 1 0.13740  335613.7  0.13740  335613.7
  7   8 1 0.13740  335613.7  0.13740  335613.7
  8   9 1 0.13740  335613.7  0.13740  335613.7
  9  10 1 0.13740  335613.7  0.13740  335613.7
 27  11 1 0.14230  329290.0  0.14230  329290.0
```

27	29	1	0.13690	351030.1	0.13690	351030.1
27	31	1	0.12220	779866.6	0.12220	779866.6
11	12	1	0.13770	428655.8	0.13770	428655.8
11	22	1	0.13640	379454.5	0.13640	379454.5
12	13	1	0.14180	259734.4	0.14180	259734.4
13	21	1	0.13350	497307.7	0.13350	497307.7
13	33	1	0.14690	272080.5	0.14690	272080.5
2	14	1	0.13640	379454.5	0.13640	379454.5
2	15	1	0.13130	501403.0	0.13130	501403.0
32	22	1	0.14450	368193.7	0.14450	368193.7
32	34	1	0.15080	256422.3	0.15080	256422.3
32	35	1	0.15080	256422.3	0.15080	256422.3
33	36	1	0.13600	361990.5	0.13600	361990.5
33	37	1	0.13600	361990.5	0.13600	361990.5
16	15	1	0.13690	268346.7	0.13690	268346.7
28	14	1	0.14450	368193.7	0.14450	368193.7
28	17	1	0.14860	298517.5	0.14860	298517.5
17	18	1	0.13740	335613.7	0.13740	335613.7
18	19	1	0.13740	335613.7	0.13740	335613.7
21	22	1	0.13390	332000.4	0.13390	332000.4
38	2	1	0.10800	333084.1	0.10800	333084.1
39	3	1	0.10840	319534.6	0.10840	319534.6
40	23	1	0.10930	287014.9	0.10930	287014.9
41	23	1	0.10930	287014.9	0.10930	287014.9
42	24	1	0.10930	287014.9	0.10930	287014.9
43	24	1	0.10930	287014.9	0.10930	287014.9
44	25	1	0.10930	287014.9	0.10930	287014.9
45	25	1	0.10930	287014.9	0.10930	287014.9
46	26	1	0.10930	287014.9	0.10930	287014.9
47	6	1	0.10840	319534.6	0.10840	319534.6
48	7	1	0.10840	319534.6	0.10840	319534.6
49	8	1	0.10840	319534.6	0.10840	319534.6
50	9	1	0.10840	319534.6	0.10840	319534.6
51	10	1	0.10840	319534.6	0.10840	319534.6
52	12	1	0.10800	331578.7	0.10800	331578.7
53	16	1	0.10800	331578.7	0.10800	331578.7
54	28	1	0.10930	287014.9	0.10930	287014.9
55	18	1	0.10840	319534.6	0.10840	319534.6
56	19	1	0.10840	319534.6	0.10840	319534.6
57	29	1	0.10150	401254.8	0.10150	401254.8
58	32	1	0.10930	287014.9	0.10930	287014.9
59	33	1	0.10930	287014.9	0.10930	287014.9
60	35	1	0.10930	287014.9	0.10930	287014.9
61	35	1	0.10930	287014.9	0.10930	287014.9

```
 62  35 1 0.10930  287014.9  0.10930  287014.9
 63  30 1 0.09720  469365.3  0.09720  469365.3
 64  34 1 0.10930  287014.9  0.10930  287014.9
 65  34 1 0.10930  287014.9  0.10930  287014.9
 66  34 1 0.10930  287014.9  0.10930  287014.9

[ pairs ]
; ai aj fu
  1  38 1
  1  17 1
  1  26 1
  1  54 1
  1  25 1
  1  42 1
  1  43 1
  2  23 1
  2  17 1
  2  26 1
  2  54 1
  2  53 1
  3   6 1
  3  10 1
  3  18 1
  3  28 1
  3  56 1
  3  27 1
  3  57 1
  4  19 1
  4  29 1
  4   7 1
  4  47 1
  4   9 1
  4  51 1
  4  55 1
  4  14 1
  4  26 1
  4  54 1
  5  20 1
  5  39 1
  5  18 1
  5  28 1
  5   8 1
  5  48 1
  5  50 1
```

6	17	1
6	9	1
6	51	1
6	49	1
7	10	1
7	50	1
8	47	1
8	51	1
9	48	1
10	17	1
10	47	1
10	49	1
11	33	1
11	34	1
11	35	1
11	58	1
11	20	1
11	57	1
12	32	1
12	29	1
12	31	1
12	36	1
12	37	1
12	59	1
13	27	1
13	32	1
14	53	1
14	24	1
14	40	1
14	41	1
14	18	1
14	25	1
14	30	1
14	46	1
15	28	1
15	23	1
16	28	1
16	24	1
16	40	1
16	41	1
16	38	1
17	20	1
17	39	1
17	56	1

17	25	1
17	30	1
17	46	1
18	26	1
18	54	1
18	29	1
19	28	1
19	39	1
19	27	1
19	57	1
20	55	1
20	31	1
21	52	1
21	36	1
21	37	1
21	59	1
21	27	1
21	34	1
21	35	1
21	58	1
22	52	1
22	29	1
22	31	1
22	33	1
22	64	1
22	65	1
22	66	1
22	60	1
22	61	1
22	62	1
23	28	1
23	53	1
23	26	1
23	44	1
23	45	1
24	28	1
24	30	1
24	46	1
25	40	1
25	41	1
25	54	1
25	63	1
26	42	1
26	43	1

**27**	**52**	**1**
**27**	**32**	**1**
**28**	**38**	**1**
**28**	**55**	**1**
**28**	**44**	**1**
**28**	**45**	**1**
**28**	**63**	**1**
**29**	**39**	**1**
**29**	**56**	**1**
**30**	**44**	**1**
**30**	**45**	**1**
**30**	**54**	**1**
**31**	**57**	**1**
**33**	**52**	**1**
**34**	**60**	**1**
**34**	**61**	**1**
**34**	**62**	**1**
**35**	**64**	**1**
**35**	**65**	**1**
**35**	**66**	**1**
**40**	**42**	**1**
**40**	**43**	**1**
**41**	**42**	**1**
**41**	**43**	**1**
**42**	**44**	**1**
**42**	**45**	**1**
**43**	**44**	**1**
**43**	**45**	**1**
**44**	**46**	**1**
**45**	**46**	**1**
**46**	**54**	**1**
**46**	**63**	**1**
**47**	**48**	**1**
**48**	**49**	**1**
**49**	**50**	**1**
**50**	**51**	**1**
**55**	**56**	**1**
**58**	**64**	**1**
**58**	**65**	**1**
**58**	**66**	**1**
**58**	**60**	**1**
**58**	**61**	**1**
**58**	**62**	**1**

```
[ angles ]
; ai aj ak fu th0 kth ub0 kub th0 kth ub0 kub
 14   1  16 1  107.2550  489.59    107.2550  489.59
 14   1  23 1  121.8320  563.07    121.8320  563.07
 16   1  23 1  131.3780  443.83    131.3780  443.83
 14   2  15 1  110.8650  609.43    110.8650  609.43
 14   2  38 1  121.1270  371.56    121.1270  371.56
 15   2  38 1  125.1340  387.22    125.1340  387.22
  4   3  20 1  119.9770  402.88    119.9770  402.88
  4   3  39 1  120.5710  339.05    120.5710  339.05
 20   3  39 1  120.5710  339.05    120.5710  339.05
  3   4   5 1  119.9770  402.88    119.9770  402.88
  3   4  17 1  119.9770  402.88    119.9770  402.88
  5   4  17 1  119.9770  402.88    119.9770  402.88
  4   5   6 1  119.9770  402.88    119.9770  402.88
  4   5  10 1  119.9770  402.88    119.9770  402.88
  6   5  10 1  119.9770  402.88    119.9770  402.88
  5   6   7 1  119.9770  402.88    119.9770  402.88
  5   6  47 1  120.5710  339.05    120.5710  339.05
  7   6  47 1  120.5710  339.05    120.5710  339.05
  6   7   8 1  119.9770  402.88    119.9770  402.88
  6   7  48 1  120.5710  339.05    120.5710  339.05
  8   7  48 1  120.5710  339.05    120.5710  339.05
  7   8   9 1  119.9770  402.88    119.9770  402.88
  7   8  49 1  120.5710  339.05    120.5710  339.05
  9   8  49 1  120.5710  339.05    120.5710  339.05
  8   9  10 1  119.9770  402.88    119.9770  402.88
  8   9  50 1  120.5710  339.05    120.5710  339.05
 10   9  50 1  120.5710  339.05    120.5710  339.05
  5  10   9 1  119.9770  402.88    119.9770  402.88
  5  10  51 1  120.5710  339.05    120.5710  339.05
  9  10  51 1  120.5710  339.05    120.5710  339.05
 12  11  22 1  107.2550  489.59    107.2550  489.59
 12  11  27 1  130.0650  461.29    130.0650  461.29
 22  11  27 1  125.3950  541.99    125.3950  541.99
 11  12  13 1  108.2390  521.51    108.2390  521.51
 11  12  52 1  126.1700  301.71    126.1700  301.71
 13  12  52 1  127.4050  328.80    127.4050  328.80
 12  13  21 1  113.5700  551.63    113.5700  551.63
 12  13  33 1  128.0610  461.29    128.0610  461.29
 21  13  33 1  120.6400  579.93    120.6400  579.93
  1  14   2 1  109.5990  693.74    109.5990  693.74
  1  14  28 1  123.3800  514.29    123.3800  514.29
  2  14  28 1  123.3800  514.29    123.3800  514.29
```

2	15	16	1	103.7790	726.27	103.7790	726.27
1	16	15	1	111.6210	625.09	111.6210	625.09
1	16	53	1	126.1700	301.71	126.1700	301.71
15	16	53	1	120.4780	420.94	120.4780	420.94
4	17	18	1	119.9770	402.88	119.9770	402.88
4	17	28	1	120.4190	483.57	120.4190	483.57
18	17	28	1	120.4190	483.57	120.4190	483.57
17	18	19	1	119.9770	402.88	119.9770	402.88
17	18	55	1	120.5710	339.05	120.5710	339.05
19	18	55	1	120.5710	339.05	120.5710	339.05
18	19	20	1	119.9770	402.88	119.9770	402.88
18	19	56	1	120.5710	339.05	120.5710	339.05
20	19	56	1	120.5710	339.05	120.5710	339.05
3	20	19	1	119.9770	402.88	119.9770	402.88
3	20	29	1	117.9180	617.27	117.9180	617.27
19	20	29	1	117.9180	617.27	117.9180	617.27
13	21	22	1	101.5500	1046.64	101.5500	1046.64
11	22	21	1	112.0870	773.24	112.0870	773.24
11	22	32	1	123.3800	514.29	123.3800	514.29
21	22	32	1	118.0490	669.06	118.0490	669.06
1	23	24	1	110.0580	605.82	110.0580	605.82
1	23	40	1	110.4670	373.97	110.4670	373.97
1	23	41	1	110.4670	373.97	110.4670	373.97
24	23	40	1	110.5490	383.00	110.5490	383.00
24	23	41	1	110.5490	383.00	110.5490	383.00
40	23	41	1	108.8360	310.74	108.8360	310.74
23	24	25	1	109.6080	512.48	109.6080	512.48
23	24	42	1	110.5490	383.00	110.5490	383.00
23	24	43	1	110.5490	383.00	110.5490	383.00
25	24	42	1	110.5490	383.00	110.5490	383.00
25	24	43	1	110.5490	383.00	110.5490	383.00
42	24	43	1	108.8360	310.74	108.8360	310.74
24	25	26	1	109.6080	512.48	109.6080	512.48
24	25	44	1	110.5490	383.00	110.5490	383.00
24	25	45	1	110.5490	383.00	110.5490	383.00
26	25	44	1	110.5490	383.00	110.5490	383.00
26	25	45	1	110.5490	383.00	110.5490	383.00
44	25	45	1	108.8360	310.74	108.8360	310.74
25	26	28	1	109.6080	512.48	109.6080	512.48
25	26	30	1	108.1330	597.39	108.1330	597.39
25	26	46	1	110.5490	383.00	110.5490	383.00
28	26	30	1	108.1330	597.39	108.1330	597.39
28	26	46	1	110.5490	383.00	110.5490	383.00
30	26	46	1	108.5770	470.32	108.5770	470.32

```
 11  27  29 1  114.6230  647.37    114.6230  647.37
 11  27  31 1  126.4560  623.88    126.4560  623.88
 29  27  31 1  127.1520  546.20    127.1520  546.20
 14  28  17 1  109.5000  602.21    109.5000  602.21
 14  28  26 1  109.1700  558.25    109.1700  558.25
 14  28  54 1  106.2990  488.39    106.2990  488.39
 17  28  26 1  108.6170  455.27    108.6170  455.27
 17  28  54 1  109.4910  377.58    109.4910  377.58
 26  28  54 1  110.5490  383.00    110.5490  383.00
 20  29  27 1  118.5960  616.06    118.5960  616.06
 20  29  57 1  118.2270  378.18    118.2270  378.18
 27  29  57 1  120.2770  346.27    120.2770  346.27
 26  30  63 1  106.5030  477.55    106.5030  477.55
 22  32  34 1  109.1700  558.25    109.1700  558.25
 22  32  35 1  109.1700  558.25    109.1700  558.25
 22  32  58 1  106.2990  488.39    106.2990  488.39
 34  32  35 1  109.6080  512.48    109.6080  512.48
 34  32  58 1  110.5490  383.00    110.5490  383.00
 35  32  58 1  110.5490  383.00    110.5490  383.00
 13  33  36 1  109.5000  602.21    109.5000  602.21
 13  33  37 1  109.5000  602.21    109.5000  602.21
 13  33  59 1  110.4570  374.58    110.4570  374.58
 36  33  37 1  106.0810  986.42    106.0810  986.42
 36  33  59 1  107.8970  526.93    107.8970  526.93
 37  33  59 1  107.8970  526.93    107.8970  526.93
 32  34  64 1  110.5490  383.00    110.5490  383.00
 32  34  65 1  110.5490  383.00    110.5490  383.00
 32  34  66 1  110.5490  383.00    110.5490  383.00
 64  34  65 1  108.8360  310.74    108.8360  310.74
 64  34  66 1  108.8360  310.74    108.8360  310.74
 65  34  66 1  108.8360  310.74    108.8360  310.74
 32  35  60 1  110.5490  383.00    110.5490  383.00
 32  35  61 1  110.5490  383.00    110.5490  383.00
 32  35  62 1  110.5490  383.00    110.5490  383.00
 60  35  61 1  108.8360  310.74    108.8360  310.74
 60  35  62 1  108.8360  310.74    108.8360  310.74
 61  35  62 1  108.8360  310.74    108.8360  310.74

[ dihedrals ]
; ai aj ak al fu phi0 kphi mult phi0 kphi mult
  1  14   2  15 9 180.00   8.3680 2   180.00   8.3680 2
  1  14   2  38 9 180.00   8.3680 2   180.00   8.3680 2
  1  14  28  26 9 180.00  -0.1674 2   180.00  -0.1674 2
  1  14  28  26 9   0.00  -0.1172 3     0.00  -0.1172 3
```

1	14	28	54	9	0.00	-0.2385	3	0.00	-0.2385	3
1	16	15	2	9	180.00	14.6440	2	180.00	14.6440	2
1	23	24	25	9	0.00	0.6276	3	0.00	0.6276	3
1	23	24	42	9	0.00	0.6276	3	0.00	0.6276	3
1	23	24	43	9	0.00	0.6276	3	0.00	0.6276	3
2	14	1	16	9	180.00	8.3680	2	180.00	8.3680	2
2	14	1	23	9	180.00	8.3680	2	180.00	8.3680	2
2	14	28	26	9	180.00	-0.1674	2	180.00	-0.1674	2
2	14	28	26	9	0.00	-0.1172	3	0.00	-0.1172	3
2	14	28	54	9	0.00	-0.2385	3	0.00	-0.2385	3
2	15	16	53	9	180.00	14.6440	2	180.00	14.6440	2
3	4	5	6	9	180.00	4.1840	2	180.00	4.1840	2
3	4	5	10	9	180.00	4.1840	2	180.00	4.1840	2
3	4	17	18	9	180.00	4.1840	2	180.00	4.1840	2
3	4	17	28	9	180.00	14.6440	2	180.00	14.6440	2
3	20	19	18	9	180.00	4.1840	2	180.00	4.1840	2
3	20	19	56	9	180.00	14.6440	2	180.00	14.6440	2
3	20	29	27	9	180.00	12.5520	2	180.00	12.5520	2
3	20	29	57	9	180.00	12.5520	2	180.00	12.5520	2
4	3	20	19	9	180.00	4.1840	2	180.00	4.1840	2
4	3	20	29	9	180.00	14.6440	2	180.00	14.6440	2
4	5	6	7	9	180.00	4.1840	2	180.00	4.1840	2
4	5	6	47	9	180.00	14.6440	2	180.00	14.6440	2
4	5	10	9	9	180.00	4.1840	2	180.00	4.1840	2
4	5	10	51	9	180.00	14.6440	2	180.00	14.6440	2
4	17	18	19	9	180.00	4.1840	2	180.00	4.1840	2
4	17	18	55	9	180.00	14.6440	2	180.00	14.6440	2
4	17	28	14	9	0.00	0.4184	3	0.00	0.4184	3
4	17	28	26	9	180.00	0.9414	2	180.00	0.9414	2
4	17	28	54	9	180.00	-0.8786	2	180.00	-0.8786	2
4	17	28	54	9	0.00	0.8201	3	0.00	0.8201	3
5	4	3	20	9	180.00	4.1840	2	180.00	4.1840	2
5	4	3	39	9	180.00	14.6440	2	180.00	14.6440	2
5	4	17	18	9	180.00	4.1840	2	180.00	4.1840	2
5	4	17	28	9	180.00	14.6440	2	180.00	14.6440	2
5	6	7	8	9	180.00	4.1840	2	180.00	4.1840	2
5	6	7	48	9	180.00	14.6440	2	180.00	14.6440	2
5	10	9	8	9	180.00	4.1840	2	180.00	4.1840	2
5	10	9	50	9	180.00	14.6440	2	180.00	14.6440	2
6	5	4	17	9	180.00	4.1840	2	180.00	4.1840	2
6	5	10	9	9	180.00	4.1840	2	180.00	4.1840	2
6	5	10	51	9	180.00	14.6440	2	180.00	14.6440	2
6	7	8	9	9	180.00	4.1840	2	180.00	4.1840	2
6	7	8	49	9	180.00	14.6440	2	180.00	14.6440	2

7	6	5	10	9	180.00	4.1840	2	180.00	4.1840	2
7	8	9	10	9	180.00	4.1840	2	180.00	4.1840	2
7	8	9	50	9	180.00	14.6440	2	180.00	14.6440	2
8	7	6	47	9	180.00	14.6440	2	180.00	14.6440	2
8	9	10	51	9	180.00	14.6440	2	180.00	14.6440	2
9	8	7	48	9	180.00	14.6440	2	180.00	14.6440	2
10	5	4	17	9	180.00	4.1840	2	180.00	4.1840	2
10	5	6	47	9	180.00	14.6440	2	180.00	14.6440	2
10	9	8	49	9	180.00	14.6440	2	180.00	14.6440	2
11	12	13	21	9	180.00	14.6440	2	180.00	14.6440	2
11	12	13	33	9	180.00	14.6440	2	180.00	14.6440	2
11	22	21	13	9	180.00	8.3680	2	180.00	8.3680	2
11	22	32	34	9	180.00	-0.1674	2	180.00	-0.1674	2
11	22	32	34	9	0.00	-0.1172	3	0.00	-0.1172	3
11	22	32	35	9	180.00	-0.1674	2	180.00	-0.1674	2
11	22	32	35	9	0.00	-0.1172	3	0.00	-0.1172	3
11	22	32	58	9	0.00	-0.2385	3	0.00	-0.2385	3
11	27	29	20	9	180.00	12.5520	2	180.00	12.5520	2
11	27	29	57	9	180.00	12.5520	2	180.00	12.5520	2
12	11	22	21	9	180.00	8.3680	2	180.00	8.3680	2
12	11	22	32	9	180.00	8.3680	2	180.00	8.3680	2
12	11	27	29	9	180.00	5.2300	2	180.00	5.2300	2
12	11	27	31	9	180.00	5.2300	2	180.00	5.2300	2
12	13	21	22	9	180.00	14.6440	2	180.00	14.6440	2
13	12	11	22	9	180.00	14.6440	2	180.00	14.6440	2
13	12	11	27	9	180.00	14.6440	2	180.00	14.6440	2
13	21	22	32	9	180.00	8.3680	2	180.00	8.3680	2
14	1	16	15	9	180.00	14.6440	2	180.00	14.6440	2
14	1	16	53	9	180.00	14.6440	2	180.00	14.6440	2
14	2	15	16	9	180.00	14.6440	2	180.00	14.6440	2
14	28	17	18	9	0.00	0.4184	3	0.00	0.4184	3
14	28	26	25	9	0.00	0.6276	3	0.00	0.6276	3
14	28	26	30	9	0.00	0.6276	3	0.00	0.6276	3
14	28	26	46	9	0.00	0.5816	3	0.00	0.5816	3
15	2	14	28	9	180.00	8.3680	2	180.00	8.3680	2
15	16	1	23	9	180.00	14.6440	2	180.00	14.6440	2
16	1	14	28	9	180.00	8.3680	2	180.00	8.3680	2
16	15	2	38	9	180.00	14.6440	2	180.00	14.6440	2
17	4	3	20	9	180.00	4.1840	2	180.00	4.1840	2
17	4	3	39	9	180.00	14.6440	2	180.00	14.6440	2
17	18	19	20	9	180.00	4.1840	2	180.00	4.1840	2
17	18	19	56	9	180.00	14.6440	2	180.00	14.6440	2
17	28	26	25	9	0.00	0.6276	3	0.00	0.6276	3
17	28	26	30	9	0.00	0.6276	3	0.00	0.6276	3

17	28	26	46	9	0.00	0.8159	3	0.00	0.8159	3
18	17	28	26	9	180.00	0.9414	2	180.00	0.9414	2
18	17	28	54	9	180.00	-0.8786	2	180.00	-0.8786	2
18	17	28	54	9	0.00	0.8201	3	0.00	0.8201	3
18	19	20	29	9	180.00	14.6440	2	180.00	14.6440	2
19	18	17	28	9	180.00	14.6440	2	180.00	14.6440	2
19	20	3	39	9	180.00	14.6440	2	180.00	14.6440	2
19	20	29	27	9	180.00	12.5520	2	180.00	12.5520	2
19	20	29	57	9	180.00	12.5520	2	180.00	12.5520	2
20	19	18	55	9	180.00	14.6440	2	180.00	14.6440	2
20	29	27	31	9	180.00	12.5520	2	180.00	12.5520	2
21	13	12	52	9	180.00	14.6440	2	180.00	14.6440	2
21	22	11	27	9	180.00	8.3680	2	180.00	8.3680	2
22	11	12	52	9	180.00	14.6440	2	180.00	14.6440	2
22	11	27	29	9	180.00	5.2300	2	180.00	5.2300	2
22	11	27	31	9	180.00	5.2300	2	180.00	5.2300	2
22	21	13	33	9	180.00	14.6440	2	180.00	14.6440	2
22	32	34	64	9	0.00	0.5816	3	0.00	0.5816	3
22	32	34	65	9	0.00	0.5816	3	0.00	0.5816	3
22	32	34	66	9	0.00	0.5816	3	0.00	0.5816	3
22	32	35	60	9	0.00	0.5816	3	0.00	0.5816	3
22	32	35	61	9	0.00	0.5816	3	0.00	0.5816	3
22	32	35	62	9	0.00	0.5816	3	0.00	0.5816	3
23	1	14	28	9	180.00	8.3680	2	180.00	8.3680	2
23	1	16	53	9	180.00	14.6440	2	180.00	14.6440	2
23	24	25	26	9	0.00	0.2134	1	0.00	0.2134	1
23	24	25	26	9	180.00	1.4267	2	180.00	1.4267	2
23	24	25	26	9	0.00	0.6945	3	0.00	0.6945	3
23	24	25	44	9	0.00	1.3389	1	0.00	1.3389	1
23	24	25	44	9	180.00	-1.3180	2	180.00	-1.3180	2
23	24	25	44	9	0.00	0.5523	3	0.00	0.5523	3
23	24	25	45	9	0.00	1.3389	1	0.00	1.3389	1
23	24	25	45	9	180.00	-1.3180	2	180.00	-1.3180	2
23	24	25	45	9	0.00	0.5523	3	0.00	0.5523	3
24	25	26	28	9	0.00	0.2134	1	0.00	0.2134	1
24	25	26	28	9	180.00	1.4267	2	180.00	1.4267	2
24	25	26	28	9	0.00	0.6945	3	0.00	0.6945	3
24	25	26	30	9	0.00	-1.4393	1	0.00	-1.4393	1
24	25	26	30	9	180.00	3.6777	2	180.00	3.6777	2
24	25	26	30	9	0.00	0.9958	3	0.00	0.9958	3
24	25	26	46	9	0.00	1.3389	1	0.00	1.3389	1
24	25	26	46	9	180.00	-1.3180	2	180.00	-1.3180	2
24	25	26	46	9	0.00	0.5523	3	0.00	0.5523	3
25	24	23	40	9	0.00	1.3389	1	0.00	1.3389	1

25 24 23 40 9 180.00 -1.3180 2 180.00 -1.3180 2
25 24 23 40 9 0.00 0.5523 3 0.00 0.5523 3
25 24 23 41 9 0.00 1.3389 1 0.00 1.3389 1
25 24 23 41 9 180.00 -1.3180 2 180.00 -1.3180 2
25 24 23 41 9 0.00 0.5523 3 0.00 0.5523 3
25 26 28 54 9 0.00 1.3389 1 0.00 1.3389 1
25 26 28 54 9 180.00 -1.3180 2 180.00 -1.3180 2
25 26 28 54 9 0.00 0.5523 3 0.00 0.5523 3
25 26 30 63 9 180.00 0.5648 2 180.00 0.5648 2
25 26 30 63 9 0.00 0.4937 3 0.00 0.4937 3
26 25 24 42 9 0.00 1.3389 1 0.00 1.3389 1
26 25 24 42 9 180.00 -1.3180 2 180.00 -1.3180 2
26 25 24 42 9 0.00 0.5523 3 0.00 0.5523 3
26 25 24 43 9 0.00 1.3389 1 0.00 1.3389 1
26 25 24 43 9 180.00 -1.3180 2 180.00 -1.3180 2
26 25 24 43 9 0.00 0.5523 3 0.00 0.5523 3
27 11 22 32 9 180.00 12.5520 2 180.00 12.5520 2
28 14 2 38 9 180.00 8.3680 2 180.00 8.3680 2
28 17 18 55 9 180.00 14.6440 2 180.00 14.6440 2
28 26 25 44 9 0.00 1.3389 1 0.00 1.3389 1
28 26 25 44 9 180.00 -1.3180 2 180.00 -1.3180 2
28 26 25 44 9 0.00 0.5523 3 0.00 0.5523 3
28 26 25 45 9 0.00 1.3389 1 0.00 1.3389 1
28 26 25 45 9 180.00 -1.3180 2 180.00 -1.3180 2
28 26 25 45 9 0.00 0.5523 3 0.00 0.5523 3
28 26 30 63 9 180.00 0.5648 2 180.00 0.5648 2
28 26 30 63 9 0.00 0.4937 3 0.00 0.4937 3
29 20 3 39 9 180.00 14.6440 2 180.00 14.6440 2
29 20 19 56 9 180.00 14.6440 2 180.00 14.6440 2
30 26 25 44 9 0.00 -1.3682 1 0.00 -1.3682 1
30 26 25 44 9 180.00 2.2426 2 180.00 2.2426 2
30 26 25 44 9 0.00 0.5858 3 0.00 0.5858 3
30 26 25 45 9 0.00 -1.3682 1 0.00 -1.3682 1
30 26 25 45 9 180.00 2.2426 2 180.00 2.2426 2
30 26 25 45 9 0.00 0.5858 3 0.00 0.5858 3
30 26 28 54 9 0.00 -1.3682 1 0.00 -1.3682 1
30 26 28 54 9 180.00 2.2426 2 180.00 2.2426 2
30 26 28 54 9 0.00 0.5858 3 0.00 0.5858 3
31 27 29 57 9 0.00 3.0041 1 0.00 3.0041 1
31 27 29 57 9 180.00 10.4056 2 180.00 10.4056 2
31 27 29 57 9 0.00 -0.9498 3 0.00 -0.9498 3
33 13 12 52 9 180.00 14.6440 2 180.00 14.6440 2
34 32 35 60 9 0.00 1.3389 1 0.00 1.3389 1
34 32 35 60 9 180.00 -1.3180 2 180.00 -1.3180 2

**34**	**32**	**35**	**60**	**9**	**0.00**	**0.5523**	**3**	**0.00**	**0.5523**	**3**
**34**	**32**	**35**	**61**	**9**	**0.00**	**1.3389**	**1**	**0.00**	**1.3389**	**1**
**34**	**32**	**35**	**61**	**9**	**180.00**	**-1.3180**	**2**	**180.00**	**-1.3180**	**2**
**34**	**32**	**35**	**61**	**9**	**0.00**	**0.5523**	**3**	**0.00**	**0.5523**	**3**
**34**	**32**	**35**	**62**	**9**	**0.00**	**1.3389**	**1**	**0.00**	**1.3389**	**1**
**34**	**32**	**35**	**62**	**9**	**180.00**	**-1.3180**	**2**	**180.00**	**-1.3180**	**2**
**34**	**32**	**35**	**62**	**9**	**0.00**	**0.5523**	**3**	**0.00**	**0.5523**	**3**
**35**	**32**	**34**	**64**	**9**	**0.00**	**1.3389**	**1**	**0.00**	**1.3389**	**1**
**35**	**32**	**34**	**64**	**9**	**180.00**	**-1.3180**	**2**	**180.00**	**-1.3180**	**2**
**35**	**32**	**34**	**64**	**9**	**0.00**	**0.5523**	**3**	**0.00**	**0.5523**	**3**
**35**	**32**	**34**	**65**	**9**	**0.00**	**1.3389**	**1**	**0.00**	**1.3389**	**1**
**35**	**32**	**34**	**65**	**9**	**180.00**	**-1.3180**	**2**	**180.00**	**-1.3180**	**2**
**35**	**32**	**34**	**65**	**9**	**0.00**	**0.5523**	**3**	**0.00**	**0.5523**	**3**
**35**	**32**	**34**	**66**	**9**	**0.00**	**1.3389**	**1**	**0.00**	**1.3389**	**1**
**35**	**32**	**34**	**66**	**9**	**180.00**	**-1.3180**	**2**	**180.00**	**-1.3180**	**2**
**35**	**32**	**34**	**66**	**9**	**0.00**	**0.5523**	**3**	**0.00**	**0.5523**	**3**
**40**	**23**	**24**	**42**	**9**	**0.00**	**0.5941**	**1**	**0.00**	**0.5941**	**1**
**40**	**23**	**24**	**42**	**9**	**180.00**	**-2.8995**	**2**	**180.00**	**-2.8995**	**2**
**40**	**23**	**24**	**42**	**9**	**0.00**	**0.6569**	**3**	**0.00**	**0.6569**	**3**
**40**	**23**	**24**	**43**	**9**	**0.00**	**0.5941**	**1**	**0.00**	**0.5941**	**1**
**40**	**23**	**24**	**43**	**9**	**180.00**	**-2.8995**	**2**	**180.00**	**-2.8995**	**2**
**40**	**23**	**24**	**43**	**9**	**0.00**	**0.6569**	**3**	**0.00**	**0.6569**	**3**
**41**	**23**	**24**	**42**	**9**	**0.00**	**0.5941**	**1**	**0.00**	**0.5941**	**1**
**41**	**23**	**24**	**42**	**9**	**180.00**	**-2.8995**	**2**	**180.00**	**-2.8995**	**2**
**41**	**23**	**24**	**42**	**9**	**0.00**	**0.6569**	**3**	**0.00**	**0.6569**	**3**
**41**	**23**	**24**	**43**	**9**	**0.00**	**0.5941**	**1**	**0.00**	**0.5941**	**1**
**41**	**23**	**24**	**43**	**9**	**180.00**	**-2.8995**	**2**	**180.00**	**-2.8995**	**2**
**41**	**23**	**24**	**43**	**9**	**0.00**	**0.6569**	**3**	**0.00**	**0.6569**	**3**
**42**	**24**	**25**	**44**	**9**	**0.00**	**0.5941**	**1**	**0.00**	**0.5941**	**1**
**42**	**24**	**25**	**44**	**9**	**180.00**	**-2.8995**	**2**	**180.00**	**-2.8995**	**2**
**42**	**24**	**25**	**44**	**9**	**0.00**	**0.6569**	**3**	**0.00**	**0.6569**	**3**
**42**	**24**	**25**	**45**	**9**	**0.00**	**0.5941**	**1**	**0.00**	**0.5941**	**1**
**42**	**24**	**25**	**45**	**9**	**180.00**	**-2.8995**	**2**	**180.00**	**-2.8995**	**2**
**42**	**24**	**25**	**45**	**9**	**0.00**	**0.6569**	**3**	**0.00**	**0.6569**	**3**
**43**	**24**	**25**	**44**	**9**	**0.00**	**0.5941**	**1**	**0.00**	**0.5941**	**1**
**43**	**24**	**25**	**44**	**9**	**180.00**	**-2.8995**	**2**	**180.00**	**-2.8995**	**2**
**43**	**24**	**25**	**44**	**9**	**0.00**	**0.6569**	**3**	**0.00**	**0.6569**	**3**
**43**	**24**	**25**	**45**	**9**	**0.00**	**0.5941**	**1**	**0.00**	**0.5941**	**1**
**43**	**24**	**25**	**45**	**9**	**180.00**	**-2.8995**	**2**	**180.00**	**-2.8995**	**2**
**43**	**24**	**25**	**45**	**9**	**0.00**	**0.6569**	**3**	**0.00**	**0.6569**	**3**
**44**	**25**	**26**	**46**	**9**	**0.00**	**0.5941**	**1**	**0.00**	**0.5941**	**1**
**44**	**25**	**26**	**46**	**9**	**180.00**	**-2.8995**	**2**	**180.00**	**-2.8995**	**2**
**44**	**25**	**26**	**46**	**9**	**0.00**	**0.6569**	**3**	**0.00**	**0.6569**	**3**
**45**	**25**	**26**	**46**	**9**	**0.00**	**0.5941**	**1**	**0.00**	**0.5941**	**1**

45 25 26 46 9 180.00 -2.8995 2 180.00 -2.8995 2
45 25 26 46 9 0.00 0.6569 3 0.00 0.6569 3
46 26 28 54 9 0.00 0.5941 1 0.00 0.5941 1
46 26 28 54 9 180.00 -2.8995 2 180.00 -2.8995 2
46 26 28 54 9 0.00 0.6569 3 0.00 0.6569 3
46 26 30 63 9 0.00 1.2468 1 0.00 1.2468 1
46 26 30 63 9 180.00 -0.5774 2 180.00 -0.5774 2
46 26 30 63 9 0.00 0.7238 3 0.00 0.7238 3
47 6 7 48 9 180.00 14.6440 2 180.00 14.6440 2
48 7 8 49 9 180.00 14.6440 2 180.00 14.6440 2
49 8 9 50 9 180.00 14.6440 2 180.00 14.6440 2
50 9 10 51 9 180.00 14.6440 2 180.00 14.6440 2
55 18 19 56 9 180.00 14.6440 2 180.00 14.6440 2
58 32 34 64 9 0.00 0.5941 1 0.00 0.5941 1
58 32 34 64 9 180.00 -2.8995 2 180.00 -2.8995 2
58 32 34 64 9 0.00 0.6569 3 0.00 0.6569 3
58 32 34 65 9 0.00 0.5941 1 0.00 0.5941 1
58 32 34 65 9 180.00 -2.8995 2 180.00 -2.8995 2
58 32 34 65 9 0.00 0.6569 3 0.00 0.6569 3
58 32 34 66 9 0.00 0.5941 1 0.00 0.5941 1
58 32 34 66 9 180.00 -2.8995 2 180.00 -2.8995 2
58 32 34 66 9 0.00 0.6569 3 0.00 0.6569 3
58 32 35 60 9 0.00 0.5941 1 0.00 0.5941 1
58 32 35 60 9 180.00 -2.8995 2 180.00 -2.8995 2
58 32 35 60 9 0.00 0.6569 3 0.00 0.6569 3
58 32 35 61 9 0.00 0.5941 1 0.00 0.5941 1
58 32 35 61 9 180.00 -2.8995 2 180.00 -2.8995 2
58 32 35 61 9 0.00 0.6569 3 0.00 0.6569 3
58 32 35 62 9 0.00 0.5941 1 0.00 0.5941 1
58 32 35 62 9 180.00 -2.8995 2 180.00 -2.8995 2
58 32 35 62 9 0.00 0.6569 3 0.00 0.6569 3

[dihedrals]
; ai aj ak al fu xi0 kxi xi0 kxi
1 23 14 16 2 0.00 30.1081 0.00 30.1081
14 28 1 2 2 0.00 7.2300 0.00 7.2300
2 15 14 38 2 0.00 40.9530 0.00 40.9530
28 17 14 26 2 0.00 0.0000 0.00 0.0000
28 26 14 54 2 0.00 0.0000 0.00 0.0000
17 18 28 4 2 0.00 24.0915 0.00 24.0915
4 3 17 5 2 0.00 21.0790 0.00 21.0790
5 10 4 6 2 0.00 21.0790 0.00 21.0790
6 7 5 47 2 0.00 9.0291 0.00 9.0291
7 8 6 48 2 0.00 9.0291 0.00 9.0291

```
  8   9   7  49 2   0.00   9.0291     0.00   9.0291
 18  19  17  55 2   0.00   9.0291     0.00   9.0291
  3  20   4  39 2   0.00   9.0291     0.00   9.0291
 23  24   1  40 2   0.00   0.0000     0.00   0.0000
 23  24   1  41 2   0.00   0.0000     0.00   0.0000
 24  25  23  42 2   0.00   0.0000     0.00   0.0000
 24  25  23  43 2   0.00   0.0000     0.00   0.0000
 20  19   3  29 2   0.00  21.0790     0.00  21.0790
 29  27  20  57 2   0.00 -12.0416     0.00 -12.0416
 27  11  29  31 2   0.00  78.2910     0.00  78.2910
 11  12  27  22 2   0.00  30.1081     0.00  30.1081
 12  13  11  52 2   0.00   3.6150     0.00   3.6150
 13  33  12  21 2   0.00  24.0915     0.00  24.0915
 26  25  28  30 2   0.00   0.0000     0.00   0.0000
 26  25  28  46 2   0.00   0.0000     0.00   0.0000
 22  21  11  32 2   0.00  12.0416     0.00  12.0416
 32  34  22  35 2   0.00   0.0000     0.00   0.0000
 32  34  22  58 2   0.00   0.0000     0.00   0.0000
 33  36  13  37 2   0.00   0.0000     0.00   0.0000
 33  36  13  59 2   0.00   0.0000     0.00   0.0000
 25  26  24  44 2   0.00   0.0000     0.00   0.0000
 25  26  24  45 2   0.00   0.0000     0.00   0.0000
  9  10   8  50 2   0.00   9.0291     0.00   9.0291
 10   9   5  51 2   0.00   9.0291     0.00   9.0291
 16  15   1  53 2   0.00  25.8990     0.00  25.8990
 19  20  18  56 2   0.00   9.0291     0.00   9.0291
 35  60  32  61 2   0.00   0.0000     0.00   0.0000
 35  60  32  62 2   0.00   0.0000     0.00   0.0000
 34  64  32  65 2   0.00   0.0000     0.00   0.0000
 34  64  32  66 2   0.00   0.0000     0.00   0.0000


#ifdef POSRES_LIGAND
[ position_restraints ]
; atom  type      fx      fy      fz
   1 1 1000 1000 1000
   2 1 1000 1000 1000
   3 1 1000 1000 1000
   4 1 1000 1000 1000
   5 1 1000 1000 1000
   6 1 1000 1000 1000
   7 1 1000 1000 1000
   8 1 1000 1000 1000
   9 1 1000 1000 1000
```

```
  10 1 1000 1000 1000
  11 1 1000 1000 1000
  12 1 1000 1000 1000
  13 1 1000 1000 1000
  14 1 1000 1000 1000
  15 1 1000 1000 1000
  16 1 1000 1000 1000
  17 1 1000 1000 1000
  18 1 1000 1000 1000
  19 1 1000 1000 1000
  20 1 1000 1000 1000
  21 1 1000 1000 1000
  22 1 1000 1000 1000
  23 1 1000 1000 1000
  24 1 1000 1000 1000
  25 1 1000 1000 1000
  26 1 1000 1000 1000
  27 1 1000 1000 1000
  28 1 1000 1000 1000
  29 1 1000 1000 1000
  30 1 1000 1000 1000
  31 1 1000 1000 1000
  32 1 1000 1000 1000
  33 1 1000 1000 1000
  34 1 1000 1000 1000
  35 1 1000 1000 1000
  36 1 1000 1000 1000
  37 1 1000 1000 1000
#endif
```
